# The peculiar properties of mitochondrial carriers of the SLC25 family

**DOI:** 10.1042/BCJ20253171

**Published:** 2025-07-24

**Authors:** Edmund R.S. Kunji, Vasiliki Mavridou, Martin S. King, Camila Cimadamore-Werthein, Stephany Jaiquel Baron, Scott A. Jones, Alannah C. King, Roger Springett, Deepak Chand, Shane M. Palmer, Denis Lacabanne, Sotiria Tavoulari, Jonathan J. Ruprecht

**Affiliations:** 1 Cambridge Biomedical Campus, MRC Mitochondrial Biology Unit, University of Cambridge, Keith Peters Building, Cambridge, CB2 0XY, U.K; 2 CellSpex, Kettering, Northamptonshire NN14 6GX, U.K; ‡ MRC Laboratory of Molecular Biology, Cambridge Biomedical Campus, Cambridge, United Kingdom; † Department of Pharmacology, University of Cambridge, Cambridge, United Kingdom; § Wellcome Sanger Institute, Wellcome Genome Campus, Hinxton, Cambridge, United Kingdom

**Keywords:** bioenergetics, mitochondria, oxidative phosphorylation, translocators, translocases, transport mechanism

## Abstract

With 53 members, the SLC25 mitochondrial carriers form the largest solute carrier family in humans. They transport a wide variety of substrates across the mitochondrial inner membrane to generate chemical energy and to supply molecules and ions for growth and maintenance of cells. They are among the smallest transporters in nature, yet they translocate some of the largest molecules without proton leak. With one exception, they are monomeric and have an unusual three-fold pseudo-symmetric structure. These carriers also have a unique transport mechanism, which is facilitated by six structural elements, meaning that all transmembrane helices move separately, but in a co-ordinated way. In addition, there are three functional elements that are an integral part of the alternating access mechanism, which opens and closes the carrier to the mitochondrial matrix or the intermembrane space. The first is a matrix gate, comprising the matrix salt bridge network and glutamine braces on transmembrane helices H1, H3 and H5. The second is a cytoplasmic gate, containing the cytoplasmic salt bridge network and tyrosine braces on transmembrane helices H2, H4 and H6. The third functional element is a single central substrate-binding site, the access to which is controlled by the opening and closing of the two gates in an alternating way. The electrostatic properties of the binding site facilitate the exchange of charged substrates across the inner membrane in the presence of a high membrane potential. Here, we discuss the extraordinary features of mitochondrial carriers, providing new insights into one of the most complex and dynamic transport mechanisms in nature.

## Introduction

**Figure 1:**
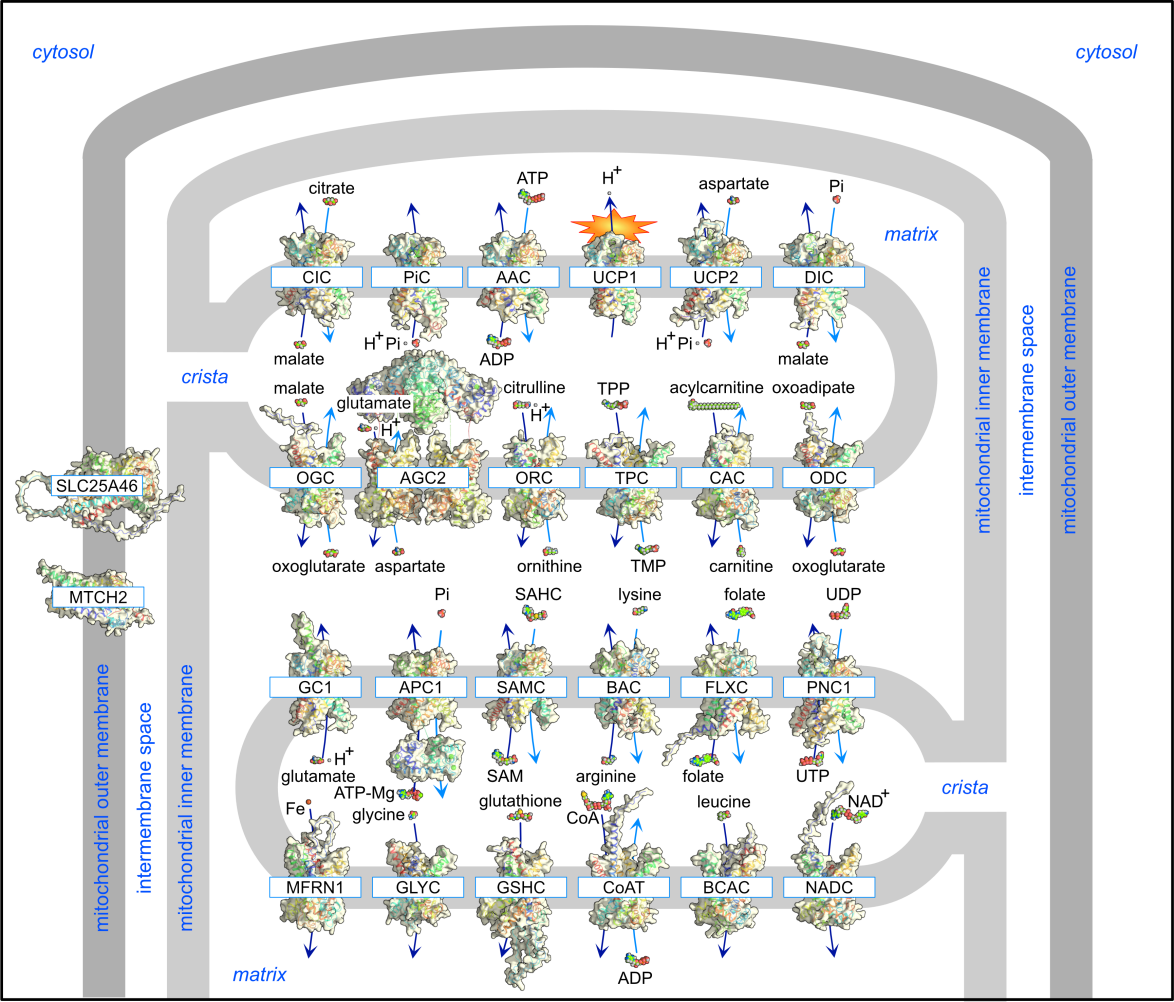
Characterised members of the human SLC25 mitochondrial family. Members of the mitochondrial carriers are shown in rainbow (blue to red) cartoon and surface representations with their primary substrates shown in sphere representations to scale. Only one paralogue is shown. The precise locations of most mitochondrial carriers in the inner membrane are unknown, but the related MTCH1 and MTCH2 as well as SLC25A46 have been proposed to be in the outer membrane. The import and export transport steps are shown in dark and light blue, respectively. The mitochondrial inner membrane is impermeable, and forms characteristic invaginations, called cristae. The membranes and compartments are labelled in blue. All models of the human carriers were determined by Alphafold 3.0 [1], except for the structures of the human uncoupling protein1 (UCP1) [2] and calcium-binding N-terminal domains of the human mitochondrial aspartate/glutamate carrier (AGC2) and ATP-Mg/Pi carrier (APC) [3,4], which were experimentally determined.

Members of the mitochondrial carrier family (solute carrier family 25, SLC25) transport a range of molecules across the impermeable inner membrane of mitochondria for many important cellular processes, such as oxidative phosphorylation driven by the degradation of fats and sugars, amino acid catabolism and interconversion, synthesis of iron sulphur clusters and haem, macromolecular synthesis, mitochondrial dynamics, apoptosis and heat production (Figure 1). There are 53 members in total, making it the largest solute carrier family in humans [5–9].

Since they are present in nearly all eukaryotes, it is highly likely that the last common eukaryotic ancestor already had a basic set of mitochondrial carriers. Over time, this set expanded, with individual carriers evolving new functions to help organisms adapt to diverse environments and survival challenges. Most of the mitochondrial carriers are transport proteins of the inner membrane (Figure 1), but there are three SLC25 members residing in the outer membrane, which are highly divergent in structure and function. The related MTCH1 (SLC25A49) and MTCH2 (SLC25A50) have a role in apoptosis, protein insertion or lipid metabolism [10–15], and SLC25A46 has a role in mitochondrial dynamics [16–18] (Figure 1). One of the most remarkable properties of the SLC25 family is the huge range of substrates that are transported by its members, varying in size from single protons to large molecules, such as S-adenosyl-methionine, palmitoyl-carnitine or nicotinamide adenine dinucleotide (Figure 1). The substrates are also highly diverse in chemical properties and include positively and negatively charged as well as polar and nonpolar molecules. Most carriers operate as strict counter-exchangers of chemically related substrates, linked by biochemical pathways, but some also display uniporter or substrate/proton symporter activities (Figure 1). Yet, as we will see later, they have the same basic fold, meaning that each carrier evolved a different substrate specificity and mode of transport within the same structural framework. Exploiting their unique properties, mitochondrial carriers play many important roles in cellular physiology. By this stage, each mitochondrial carrier probably warrants their own review, for example for SLC25A20 [19] or SLC25A13 [20], or as a subgroup [21]. Thus, in the next sections, we will focus only on the discovery of their primary transport function and regulation, and their role in cellular physiology.

## Fundamental roles of mitochondrial carriers in energy metabolism

**Figure 2:**
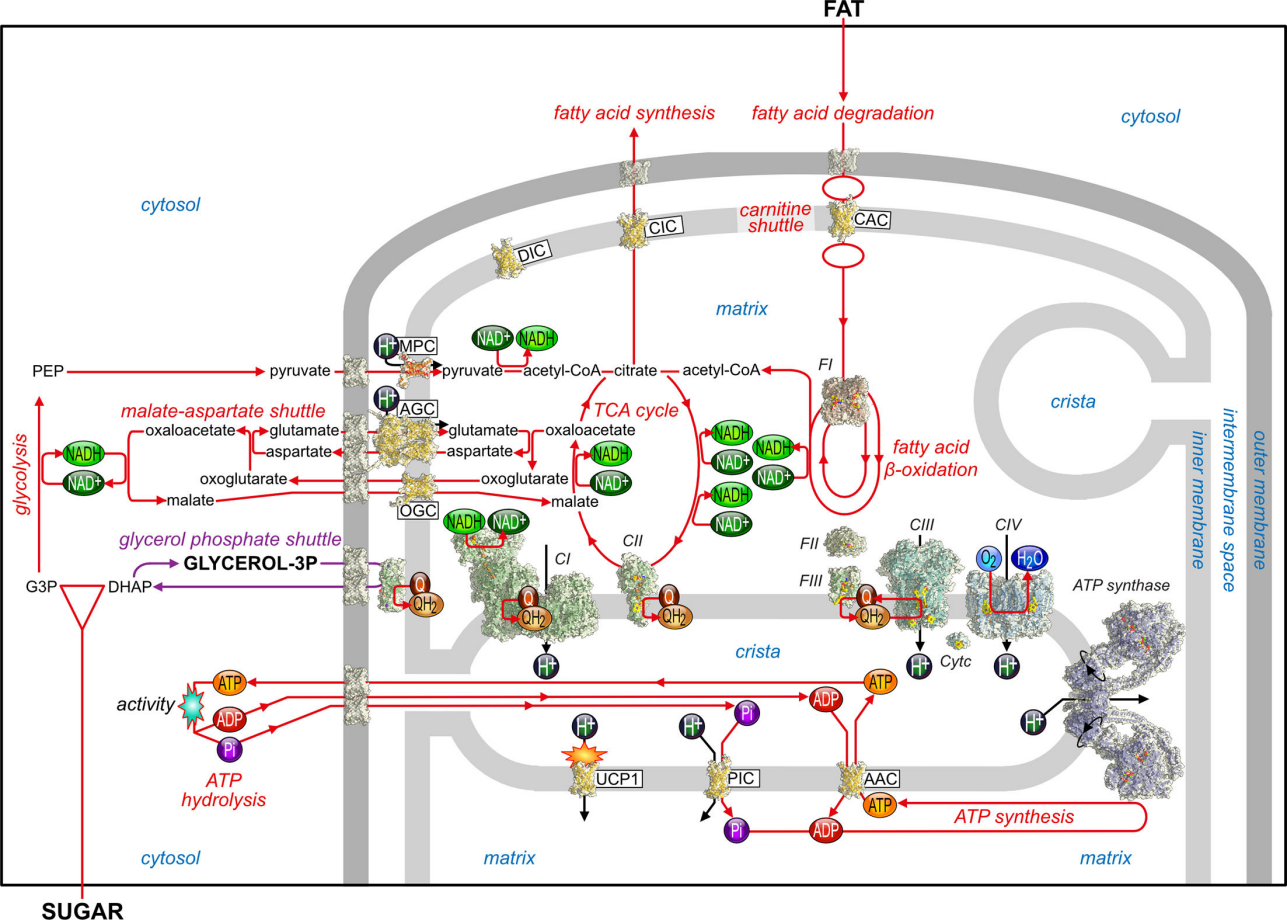
**Role of mitochondrial carriers in oxidative phosphorylation and energy metabolism**. The SLC25 mitochondrial carriers (yellow) and SLC54 mitochondrial pyruvate carrier (orange/red) play essential roles in energy metabolism from the breakdown of sugars and fat. The mitochondrial outer membrane is permeable to molecules through voltage-dependent anion channels (grey) [22], whereas the mitochondrial inner membrane is impermeable, forms characteristic invaginations, called cristae, and contains the complexes of the respiratory chain (green) [23–32] and ATP synthase (blue) [33]. All carrier models were determined by Alphafold 3.0 [1], except for the human uncoupling protein1 (SLC25A7, UCP1) [2,34] and calcium-binding N-terminal domains of the human mitochondrial aspartate/glutamate carrier (AGC) and ATP-Mg/Pi carrier (APC) [3,4]. The pathway reactions and transport steps are shown in red and the proton translocation steps in black arrows. Q, coenzyme Q; CoA, coenzyme A; CI, CII, CIII and CIV, complexes I, II, III and IV, respectively; FI, FII and FIII, long or short chain dehydrogenases, flavin transfer protein and flavin transfer protein dehydrogenase, respectively. The membranes and compartments are labelled in blue.

Sugars and fats are two major sources of metabolic energy used by human cells, which must be oxidised to generate ATP through a process called oxidative phosphorylation. Mitochondrial transport proteins are often omitted from diagrams of mitochondrial oxidative phosphorylation, even though without them there would be no import of substrates to oxidise, no ADP or phosphate to synthesise ATP and no export of ATP to the rest of the cell. In this section, we will briefly discuss the human mitochondrial carriers that play key roles in energy metabolism (Figure 2).

### Role of transport in energy production from sugar molecules

Glycolysis is the main pathway for the breakdown of sugar molecules in the cytosol, which generates two pyruvate, two ATP and two NADH molecules per glucose molecule (Figure 2). Pyruvate is then transported into the matrix by the mitochondrial pyruvate carrier, as a substrate for further degradation, leading to oxidative phosphorylation [35–38]. The mitochondrial pyruvate carrier belongs to a different transporter family, called SLC54, and is a heterodimer of two small homologous membrane proteins, each comprising an amphipathic helix and three transmembrane helices [39–42]. After import, pyruvate is converted to acetyl-coenzyme A (acetyl-CoA) by pyruvate dehydrogenase, transferring two electrons and a proton to NAD^+^ to form NADH. Acetyl-CoA then enters the tricarboxylic acid (TCA) or Krebs cycle, which leads to the production of one GTP (or ATP, depending on the tissue) and three additional NADH molecules per pyruvate molecule (Figure 2). The oxidation of NADH by complex I and succinate by complex II allows the entry of the extracted electrons into the respiratory chain to complex III, cytochrome c and complex IV, which leads to oxygen being reduced to water. The redox reactions in complexes I, III and IV are coupled to the translocation of protons from the mitochondrial matrix to the intermembrane space, generating a proton motive force [23–27], which is used by ATP synthase to drive the synthesis of ATP from ADP and inorganic phosphate via a rotary mechanism [33]. However, ATP synthesis would not be possible without two critical transport activities (Figure 2). The mitochondrial phosphate carrier (SLC25A3) imports inorganic phosphate [43,44] together with a proton [45] (Figure 2). The ΔpH of ~0.4 units is used to maintain high phosphate concentrations in the mitochondrial matrix to facilitate ATP synthesis, giving 16.8 mM and 3.3 mM for the mitochondrial and cytosolic phosphate concentration in hepatocytes, respectively [46]. The other critical transport step is carried out by mitochondrial ADP/ATP carriers (AAC), also called adenine nucleotide translocases (ANT) [47–51], which import ADP into the matrix for the synthesis of ATP [33,52] and export ATP to fuel the cell (Figure 2). In humans, there are four isoforms of the mitochondrial ADP/ATP carrier [53], AAC1 (SLC25A4) [54,55], AAC2 (SLC25A5) [56], AAC3 (SLC25A6) [55] and AAC4 (SLC25A31) [57]. The exchange of ADP for ATP is electrogenic, and the membrane potential component of the proton motive force maintains a higher ADP/ATP ratio in the matrix than the cytosol. Together, the phosphate and ADP/ATP carriers increase the phosphorylation potential of ATP in the cytosol over what can be achieved by ATP synthase alone [46].

There are many other mitochondrial carriers that deliver molecules into the TCA cycle at different entry points. The mitochondrial dicarboxylate carrier DIC (SLC25A10) transports malonate, malate, succinate, sulphate, thiosulphate and phosphate [58–61] and is involved in gluconeogenesis, ureagenesis, metabolism of sulphur compounds and in fatty acid synthesis [62]. The mitochondrial tricarboxylate or citrate carrier CIC (SLC25A1) exchanges tricarboxylates for other tricarboxylates, dicarboxylates or phosphoenolpyruvate and plays an important role in supplying intermediates for the TCA cycle and in exporting citrate as a precursor for lipid and sterol synthesis in the cytosol [63–65]. The mitochondrial oxoglutarate carrier OGC (SLC25A11) exchanges cytosolic malate for 2-oxoglutarate from the mitochondrial matrix and plays an important role in the malate-aspartate shuttle, the oxoglutarate-isocitrate shuttle and gluconeogenesis [61,66–69]. The mitochondrial oxodicarboxylate carrier ODC (SLC25A21) imports 2-oxoadipate and exports 2-oxoglutarate, playing a central role in the catabolism of lysine, hydroxylysine and tryptophan [70]. There are several mitochondrial carriers, such as UCP2 (SLC25A8) [71], UCP3 (SLC25A9) [72,73], UCP5 (SLC25A14) and UCP6 (SLC25A30) [74], which are likely to be transporters of dicarboxylic acids [75], as well as sulphate, sulphite and thiosulphate. They are related to the uncoupling protein UCP1 (SLC25A7) [2,34,76–78], which will be discussed later, even though it is still unclear whether they have an uncoupling function too.

### Role of transport in the malate-aspartate shuttle

The malate-aspartate shuttle [79,80] is critical for energy metabolism by coupling cytosolic NADH oxidation to matrix NAD^+^ reduction as well as for gluconeogenesis [81] and the urea cycle [82]. NADH produced by glycolysis in the cytosol (Figure 2) must be re-oxidised to NAD^+^; otherwise, glycolysis would halt and lactic acid would be produced through anaerobic respiration. The malate-aspartate shuttle [79] uses a series of enzymatic reactions and transport steps to oxidise NADH to NAD^+^ in the cytosol and to reduce NAD^+^ to NADH in the mitochondrial matrix, which then passes its electrons to complex I (Figure 2). Two different transport activities are involved, one mediated by the aforementioned mitochondrial oxoglutarate carrier (SLC25A11) [61,66–69] and the other by the mitochondrial aspartate/glutamate carriers (SLC25A12 and SLC25A13), which are also called AGC1/aralar and AGC2/citrin, respectively [83,84]. These carriers import glutamate with a proton and export aspartate from the mitochondrial matrix [84–87] (Figure 2). They have an unusual three-domain structure, consisting of (i) an N-terminal calcium-binding domain, (ii) a carrier domain and (iii) a C-terminal amphipathic helix, and they are the only members of the SLC25 family to form structural homodimers [3]. The consequence of coupling the malate-aspartate shuttle to the import of a proton by the aspartate/glutamate carriers is that the pathway is driven in the forward direction by the proton motive force, allowing the cytosolic NAD+/NADH pool to be maintained far more oxidised than the matrix pool [88]. Several studies have shown that the malate-aspartate shuttle is activated by calcium, a property attributed to the mitochondrial aspartate/glutamate carriers [89–93]. Their transport activities have been proposed to be regulated by calcium [83,94], but this notion is not supported by recent transport data [84] nor by structural analyses, as the N-terminal domain only has a single preformed calcium-binding EF-hand, meaning conformational changes cannot be induced by the removal of calcium [3]. In the absence of the C-terminal amphipathic helix, calcium-dependent conformational changes of the N-terminal domain of aralar (SLC25A12) are observed [3], but this is an unlikely scenario in the context of the whole complex [84]. A possible explanation for the calcium regulation of the malate-aspartate shuttle is that the conversion of malate to oxaloacetate is a critical part of the TCA cycle, which itself is calcium regulated [95,96].

An alternative pathway, called the glycerol phosphate shuttle (Figure 2), can also be used for the conversion of cytosolic NADH [97]. Here, NADH is converted to NAD^+^ by the conversion of dihydroxyacetone-phosphate to glycerol-3-phosphate. The latter is converted back to dihydroxyacetone-phosphate by glycerol-3-phosphate dehydrogenase, which is associated with the outer face of the mitochondrial inner membrane, donating electrons and protons to ubiquinone to form ubiquinol, which is oxidised by complex III (Figure 2). The regenerated dihydroxyacetone-phosphate then enters the “pay-off” phase of glycolysis to generate ATP.

### Role of mitochondrial transport in the breakdown of fat molecules

Dietary and stored lipids are hydrolysed in adipocytes to release fatty acids and glycerol, which circulate in the bloodstream. After uptake by the cell, fatty acids are activated by coupling to CoA in the cytosol. In the carnitine shuttle, fatty acid chains are transferred to carnitine by the carnitine palmitoyltransferase I, located on the mitochondrial outer membrane (Figure 2). The carnitine/acylcarnitine carrier CAC (SLC25A20) [98–100] then transports acylcarnitine into the mitochondrial matrix in exchange for free carnitine, which is generated when the fatty acid chains are transferred back onto matrix CoA by carnitine palmitoyltransferase II, located on the mitochondrial inner membrane (Figure 2). Fatty acid β-oxidation removes two carbon atoms in each cycle as acetyl-CoA, which enters the TCA cycle (Figure 2). The electrons are released from the bonds by acyl-CoA dehydrogenases, which transfer them via electron transfer flavoprotein (ETF) [28] and ETF-ubiquinone oxidoreductase [29] to ubiquinone, and by β-hydroxyacyl CoA dehydrogenases to NADH, which passes the electrons to complex I and ubiquinone (Figure 2). As with the breakdown of sugars, the transfer of electrons down the respiratory chain is coupled to the generation of a proton motive force, which is used for the synthesis of ATP (Figure 2) [33]. The lipid-derived head group choline is transported into mitochondria by the mitochondrial choline transporter (SLC25A48) for one-carbon metabolism, serving as a methyl donor, and for the production of betaine [101–106].

### Amino acids in energy generation

The breakdown of amino acids, derived from protein, can also be used for the generation of ATP during starvation, but with much lower yields. Several mitochondrial carriers play a key role in this process. The oxodicarboxylate carrier ODC (SLC25A21) imports 2-oxoadipate and exports 2-oxoglutarate, playing a central role in the catabolism of lysine, hydroxylysine and tryptophan [70,107]. Branch chain amino acids are another source of metabolic energy, which are transported into mitochondria by the mitochondrial branch chain amino acid carrier (SLC25A44) [108].

### To couple or to uncouple

For the energy conversion to work well, mitochondria need to be tightly coupled [109]. However, a mitochondrial carrier, called the uncoupling protein (SLC25A7) [76,78], plays a crucial role in mitochondrial uncoupling, where the chemical energy stored in food components is released as heat in a specialised adipose tissue, called brown fat [76,77,110]. When activated by fatty acids, the uncoupling protein dissipates the proton motive force, leading to heat production by short-circuiting the mitochondrion (Figure 1). This process bypasses ATP synthase, as the proton motive force is no longer coupled to ATP synthesis. Recently, the atomic structure of UCP1 has been solved [2,34,111], confirming that the protein is monomeric and binds three cardiolipin molecules [112]. The molecular basis of the pH-dependent binding of purine nucleotides [2] and pyrimidine nucleotides [34] has been elucidated, but the activation mechanism remains unresolved [113,114]. UCP1 has retained all of the key structural and functional features of mitochondrial carriers, suggesting that it might have a conventional carrier-like activation mechanism [2,113,115], but it has not been confirmed experimentally.

In conclusion, no aspect of mitochondrial energy metabolism would occur in the absence of these key transport steps. Thus, mitochondrial oxidative phosphorylation is not initiated by complex I, but by the mitochondrial pyruvate carrier (SLC54) [35,36,39–41] and the carnitine/acylcarnitine carrier (SLC25A20) [98–100]. Also, the final step is not ATP synthesis, but the export of ATP by the mitochondrial ADP/ATP carriers (SLC25A4, SLC25A5, SLC25A6, SLC25A31) [5,54–57,116,117] or the heat production by the uncoupling protein (SLC25A7) [2,112,113,115].

## Key roles of mitochondrial carriers in homeostasis and maintenance

Many mitochondrial transport processes are also essential for maintaining mitochondrial and cellular homeostasis and for providing building blocks for growth, which we will describe next.

### Amino acid metabolism

Mitochondria are central to the synthesis, catabolism and interconversion of amino acids, which are also used for mitochondrial protein synthesis. Humans have a limited ability to synthesise amino acids, and thus, a large number need to be taken up via the diet. The branched-chain amino acid carrier (SLC25A44), which transports valine, leucine and isoleucine into mitochondria, is central to their use in energy metabolism and thermogenesis [108] or for protein synthesis. The mitochondrial glycine carrier (SLC25A38) is involved in the import of glycine into the mitochondrial matrix, where it can be used for gluconeogenesis, protein synthesis or the synthesis of aminolevulinic acid, a precursor for haem synthesis [118]. However, SLC25A38 has also been proposed to be a transporter of pyridoxal 5'-phosphate [119], which is a co-factor used in haem synthesis. There is also a basic amino acid carrier BAC (SLC25A29), which transports arginine, lysine, homoarginine, methylarginine and, to a much lesser extent, ornithine and histidine [120]. The mitochondrial ornithine carriers (SLC25A2 and SLC25A15) catalyse the exchange of ornithine and citrulline, which links the fixation of ammonia, derived from the degradation of amino acids in the mitochondrion, to the urea cycle in the cytosol [121,122]. The mitochondrial glutamate carriers, GHC1 (SLC25A22) and GHC2 (SLC25A18), import glutamate together with a proton, but they do not export aspartate [123], unlike the previously mentioned mitochondrial aspartate/glutamate carriers (SLC25A12 and SLC25A13) [83], and they also lack the N-terminal calcium-binding domain. Although not belonging to the SLC25 family, a variant of SLC1A5 functions as a mitochondrial glutamine transporter, which plays a role in metabolic reprogramming in cancer cells [124].

### Cofactor and vitamin transport

The human thiamine pyrophosphate transporter (SLC25A19) is involved in the transport of thiamine pyrophosphate, an important co-factor in dehydrogenase reactions [125]. The mitochondrial S-adenosylmethionine carrier SAMC (SLC25A26) imports S-adenosylmethionine into mitochondria, which is required for the methylation of DNA, RNA and proteins in the mitochondrial matrix, and exports the product S-adenosylhomocysteine [126]. The transport of folate, involved in one-carbon metabolism, and the transport of flavin nucleotides (FMN/FAD), providing co-factors for electron transfer, have both been assigned to the mitochondrial carrier FLXC (SLC25A32) [127,128]. The human carriers SLC25A51 and SLC25A52 have been proposed to import NAD^+^ based on metabolic and other analyses [129–132]. Furthermore, SLC25A39, and possibly also SLC25A40, has been implicated in glutathione import (GSHC), which plays a key role in redox homeostasis [133–135]. Coenzyme A, which is required for dehydrogenase reactions, is transported into the mitochondrial matrix by the mitochondrial CoA transporter (SLC25A42) [136]. As mentioned before, SLC25A38 is likely to function as a pyridoxal 5'-phosphate transporter, another key co-factor [119].

### Nucleotide transport

The mitochondrial ADP/ATP carriers can only exchange ADP and ATP in equimolar amounts [49,50,53,137,138], meaning that the total mitochondrial adenine nucleotide pool remains unaltered. The mitochondrial ATP-Mg/Pi carriers (SLC25A23, SLC25A24, SLC25A25 and SLC25A41) exchange Mg-bound or free adenine nucleotides for phosphate [139–141]. In this way, they facilitate the net import or export of adenine nucleotides [139,140,142], altering the adenine nucleotide pool size in response to changes in energetic demand [140,142,143]. Their transport activities surge with increasing amounts of calcium, in concert with the calcium-mediated activation of the TCA enzymes [95,96]. The mitochondrial ATP-Mg/Pi carriers consist of three domains: (i) an N-terminal calcium-regulatory domain with four calcium-binding EF-hands, (ii) an amphipathic helix and (iii) a C-terminal carrier domain, and they function as monomers [4]. In the presence of calcium, the amphipathic helix is bound to the regulatory domain, allowing transport by the carrier domain to occur [4,144]. In the absence of calcium, the amphipathic helix is released from the regulatory domain and binds to the carrier domain, leading to inhibition of transport [145,146]. SLC25A41 lacks both the N-terminal calcium-regulatory domain and the amphipathic helix [141], meaning that its transport activity is not regulated by calcium.

Mitochondrial DNA replication and transcription also require the import of pyrimidine and other purine nucleotides. There are two mitochondrial pyrimidine nucleotide carriers (PNC1 and PNC2) in humans (SLC25A33 and SLC25A36), which supply nucleotides for mitochondrial DNA and RNA synthesis and breakdown [147,148]. SLC25A33 (PNC1) imports uracil, thymine and cytosine (deoxy)nucleoside di- and tri-phosphates, whereas SLC25A36 (PNC2) transports both cytosine and uracil (deoxy)nucleoside mono-, di- and tri-phosphates [147,148]. To date, no dedicated mitochondrial guanine nucleotide carrier has been identified in human mitochondria.

### Ion transport and homeostasis

The mitoferrins SLC25A37 and SLC25A28 (MFRN1 and MFRN2) have been proposed to transport iron ions into mitochondria for incorporation into haem and iron-sulphur clusters, as well as other potential functions [149,150]. The mitochondrial ATP-Mg/Pi carriers are involved in the transfer of Mg^2+^ into mitochondria [151]. It has also been suggested that Mtm1p, a yeast homologue of the human glutathione carriers SLC25A39 and SLC2540, can transport Mn^2+^ ions [152].

This round-up of known functions of human mitochondrial carriers provides just a snapshot of the current state of knowledge, and a lot of research is still aimed at clarifying the role of unidentified carriers in human physiology and disease, such as SLC25A34 [153], SLC25A35, SLC25A43 [154], SLC25A45, SLC25A47 and SLC25A53 [155]. In addition, there are other transport protein families in mitochondria, such as ATP-Binding Cassette (ABC) transporters [156,157], mitochondrial glutamine transporter (SLC1A5) [124] and sideroflexins [158–163], which also play an important, but not fully understood, role in cellular physiology.

## The unusual architecture of mitochondrial carriers

**Figure 3:**
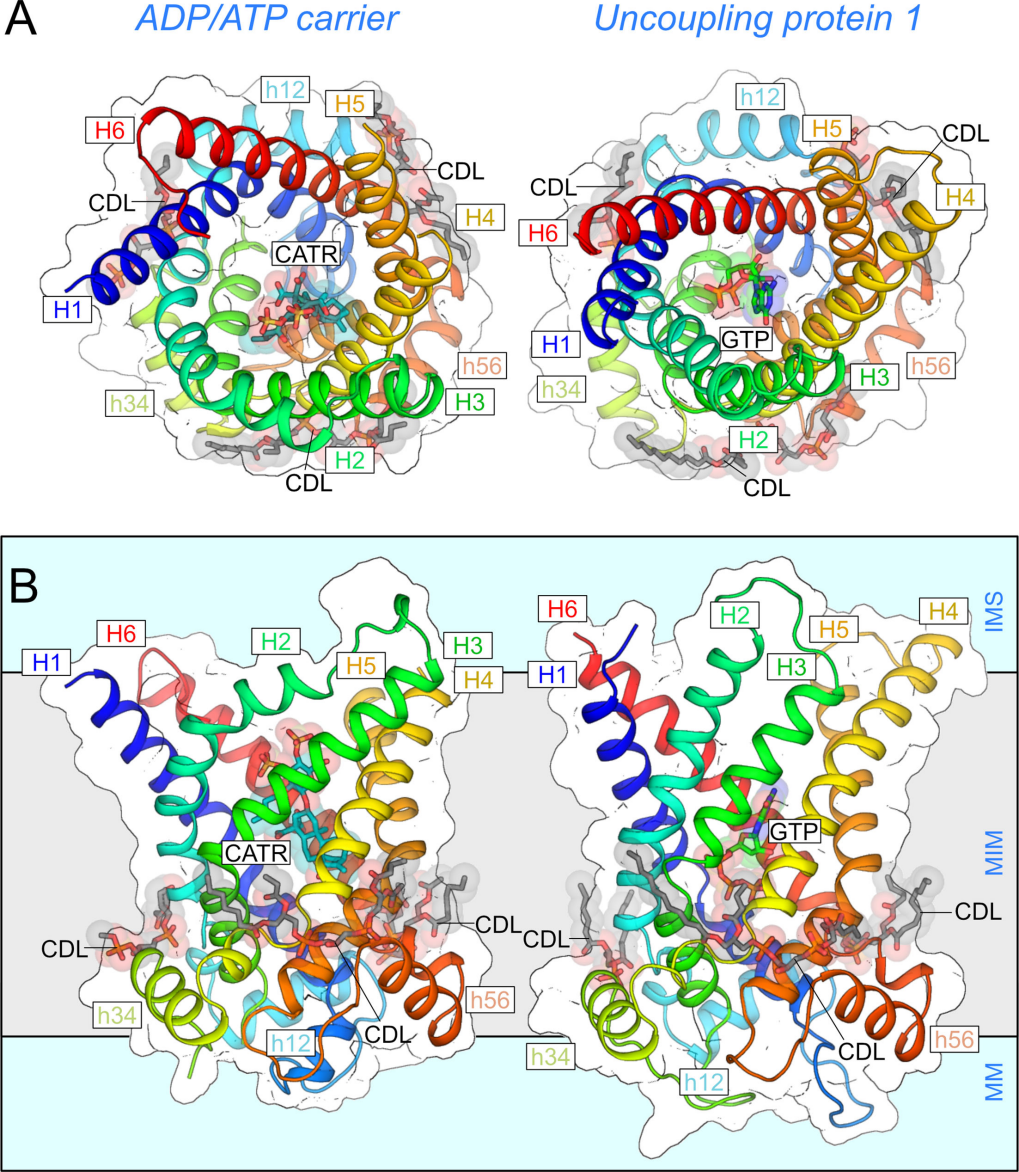
Structures of the mitochondrial ADP/ATP carrier and uncoupling protein. (**A**) Cytoplasmic and (**B**) lateral view of the mitochondrial ADP/ATP carrier (Aac2p, PDB: 4C9H) [167] (left) and the uncoupling protein (SLC25A7, PDB: 8G8W) [2](right). The structures are shown in cartoon representation using a rainbow colour scheme from the N-terminus in blue to the C-terminus in red. The inhibitors carboxy-atractyloside (CATR), GTP and cardiolipin molecules (CDL) are shown in teal, green and grey, respectively. The transmembrane and matrix helices are indicated. IMS, intermembrane space; MIM, mitochondrial inner membrane; MM, mitochondrial matrix.

The amino acid sequences of SLC25 mitochondrial carriers contain a striking repeat of three homologous sequences, each about 100 amino acids long [164], which was recognised after the first sequence became available [54]. The first structural information was obtained for the yeast ADP/ATP carrier Aac3p in complex with atractyloside, demonstrating that the carrier is a structural monomer with three-fold pseudo-symmetry in agreement with the three sequence repeats [165]. The structure redefined the topology of the six transmembrane and matrix helices and revealed a central translocation pathway for adenine nucleotides, supporting the idea that the carrier could function as a monomer [165]. The first determined atomic structure was of the bovine ADP/ATP carrier, locked in the cytoplasmic-open state by carboxy-atractyloside [166]. In this state, the central water-filled cavity is open to the intermembrane space, which is continuous with the cytosol via voltage-dependent anion channels. Later, this basic structural fold was confirmed by the atomic structures of the yeast isoforms Aac2p and Aac3p, also in the cytoplasmic-open state (Figure 3A) [167]. Subsequently, the first atomic structure of a fungal ADP/ATP carrier, locked by bongkrekic acid in the matrix-open state, was solved in which the central water-filled cavity is accessible from the mitochondrial matrix [168]. There are structures of inactive mitochondrial oxoglutarate-like carriers, which serve as structural components in the respiratory chain of Tetrahymena [169]. Recently, atomic structures have also become available of the human uncoupling protein in the cytoplasmic-open state (Figure 3) [2,34,111]. Finally, structural information is also available for the calcium-binding N-terminal domains of the mitochondrial aspartate/glutamate carriers (SLC25A12 and SLC25A13) [3] and ATP-Mg/Pi carriers (SLC25A24) [4,170].

**Figure 4:**
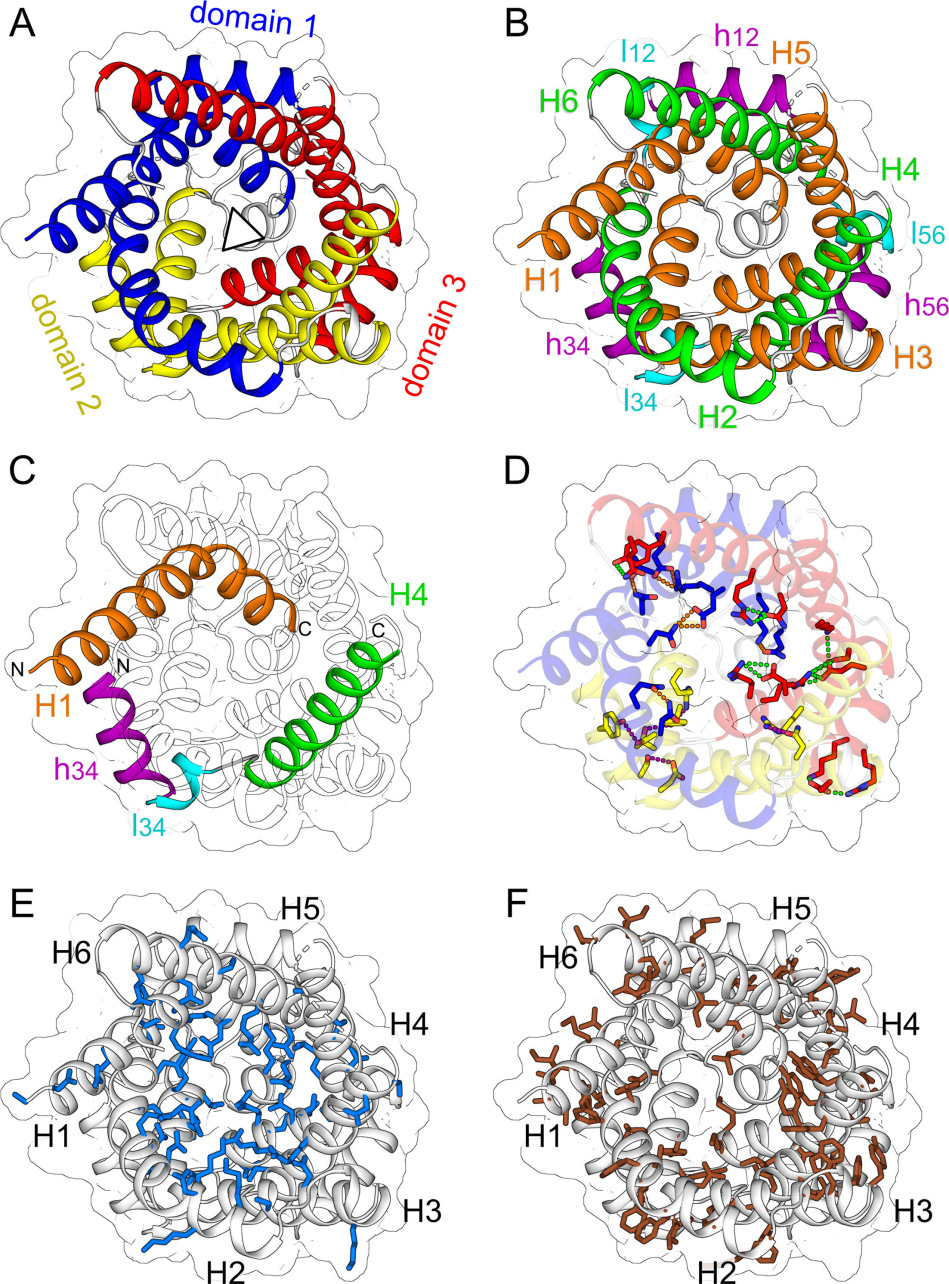
The structural features of SLC25 mitochondrial carriers. (**A**) The three-domain structure, where domains 1, 2 and 3 are coloured in blue, yellow and red respectively. (**B**) Odd-numbered transmembrane helices (**H1, H3 and H5**), matrix helices (**h12, h34 and h56**), linker helices (**l12, l34 and l56**) and even-numbered transmembrane helices (**H2, H4 and H6**) are coloured orange, magenta, cyan and green, respectively. (**C**) The path of transmembrane helix H1 (orange), matrix helix (h34, magenta), linker helix (l34, cyan) and transmembrane helix H4 (green). (**D**) Residues involved in intra-domain interactions of domain 1 (blue), domain 2 (yellow) and domain 3 (red), where the interactions are shown in orange, magenta and green, respectively. (**E**) Polar residues in the water-filled cavity and (**F**) non-polar residues interacting with the hydrophobic core of the membrane. The shown structure is the cytoplasmic view of the mitochondrial ADP/ATP carrier (PDB: 4C9H) [167].

The structure of mitochondrial carriers has several unusual features. First of all, they are composed of three domains that look similar but are not identical [165], reflecting the three homologous sequence repeats [164] (Figure 4A). The domains are arranged around a three-fold pseudo-symmetrical axis, which runs through the centre of the carrier and is approximately perpendicular to the membrane plane. Each domain consists of an odd-numbered transmembrane helix, a loop with a short matrix helix parallel to the plane of the membrane, a linker helix and an even-numbered transmembrane helix (Figure 4B) [166]. Remarkably, all transmembrane helices run approximately at 45 degree angles to the membrane plane. This unusual arrangement stabilises the helical bundle by preventing vertical movements, while allowing horizontal sliding movements of the helices across their surfaces [167]. The odd-numbered helices have peculiar L-shapes because of the presence of conserved proline or serine residues [166,167], which break the hydrogen bonding of the helix backbones (Figure 4C). The kinks are about 50 degrees and are stabilised by intra-domain interactions on the matrix side of the carrier [167]. The matrix helices lie on the surface in the head group region of the inner membrane [171–173] and are connected to the even-numbered helices via short linker helices [167]. Finally, these helices are all connected via loops, which are variable in length and composition (Figure 4C). The transmembrane helices nearly reach the other side of the carrier because of their long length, high tilt angles and curvatures, providing further stabilisation of the overall structure (Figure 4D). Each domain may contain several intrahelical and intra-domain interactions, mainly on the matrix side, but there are no significant polar interactions between the transmembrane helices, allowing them all to move relatively to each other, which is a highly unusual feature for a membrane protein. Another remarkable feature is the presence of three tightly bound cardiolipin molecules, which link the matrix and even-numbered helices across the domain interface [166,167,174]. Another unusual feature of mitochondrial carriers is that all transmembrane helices are strongly amphipathic with a hydrophilic side, facing the central water-filled cavity (Figure 4E), and a hydrophobic side, oriented towards the core of the lipid bilayer (Figure 4F). In other transport proteins, domains consist of three or more transmembrane helices that interact with each other, the aqueous phase or the membrane, and therefore do not exhibit the same degree of amphipathicity.

## The unique transport mechanism of mitochondrial carriers

**Figure 5:**
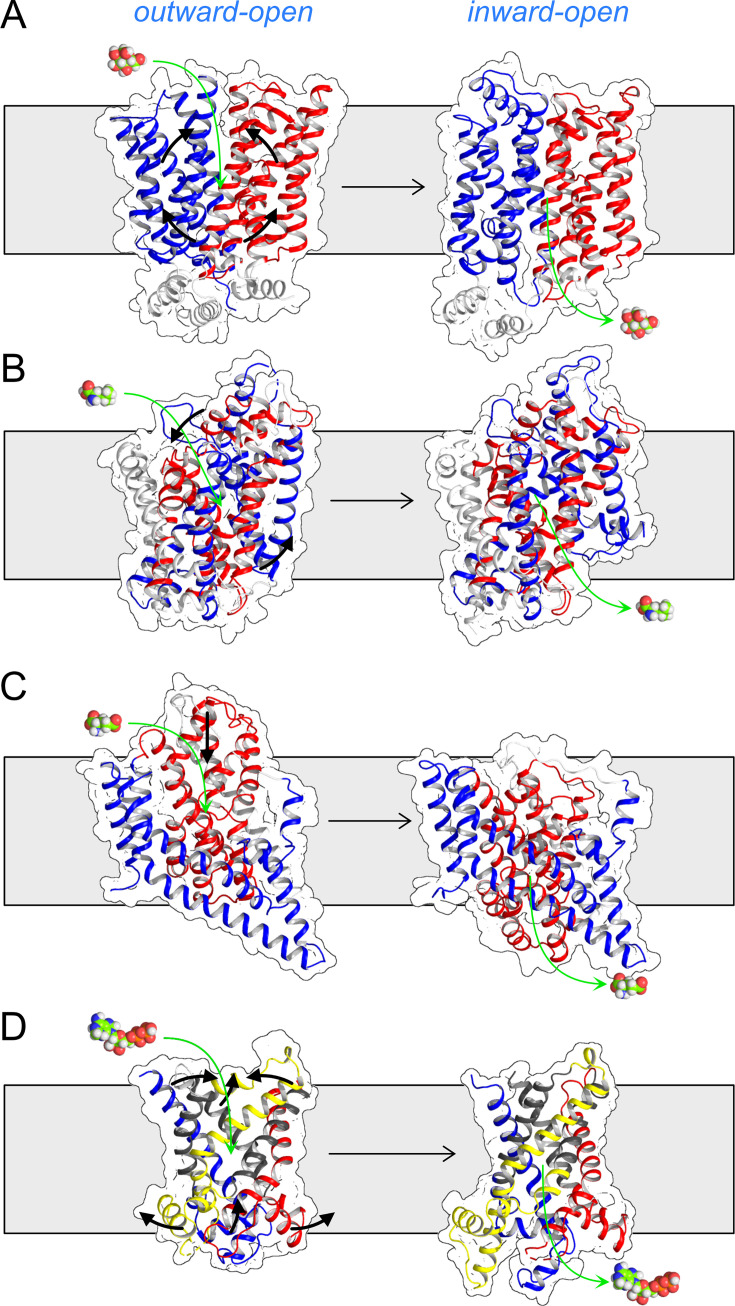
Mitochondrial carriers have a unique transport mechanism. Different alternating access mechanisms in lateral views from the membrane.(**A**) Two-domain ‘rocker-switch’ mechanism, glucose transporter Glut5 (PDB: 4YBQ and 4YB9). (**B**) Two-domain ‘rocking bundle’ mechanism, leucine transporter LeuT (PDB: 3TT1 and 3TT3). (**C**) Two-domain ‘elevator mechanism’, glutamate transporter GltP (PDB: 2NWL and 3KBC), which are all examples of a two-domain mechanism. (**D**) Three-domain ‘mitochondrial carrier’ mechanism, mitochondrial ADP/ATP carrier (PDB: 4CGH and 6CGI), which has six moving elements: three core elements (primary colours) and three gate elements (grey). The substrates are shown in sphere representations. Black arrows show the approximate protein movements, whereas the green arrows show the substrate movements.

Given the unusual architecture of SLC25 mitochondrial carriers, it was always to be expected that the transport mechanism would differ from those of other transport proteins. Other solute transport proteins have a two-domain structure [175], and thus far, three different transport mechanisms have been discerned for them. In the first, the two domains have the same topology, such as Major Facilitator Superfamily (MFS) transporters [176,177], ABC transporters [178], semiSWEET transporters [179], mitochondrial pyruvate carriers [39], and they operate with a rocker-switch mechanism (Figure 5A). In the second, the two domains have an inverted topology, such as Neurotransmitter–Sodium Symporters (NSS), which have a rocking-bundle mechanism (Figure 5B) [180–183]. The movements in both mechanisms require a rotation of the two domains around a central substrate-binding site, making it alternately accessible. The third transport mechanism, termed the elevator mechanism, involves a near vertical displacement of one domain against another domain, exposing a substrate-binding site from one side of the membrane to the other, such as the glutamate or aspartate transporters [184,185], citrate transporter [186] or sodium proton antiporter [187] (Figure 5C).

In contrast, mitochondrial carriers have a three-domain structure [165] and operate via a distinct fourth transport mechanism, unrelated to the other three. The mechanism involves three homologous domains organised around a central translocation pathway [165], with each domain containing two separately moving elements (Figure 5D) [5,6,168]. As we will see next, this complex transport mechanism has many distinctive features.

**Figure 6:**
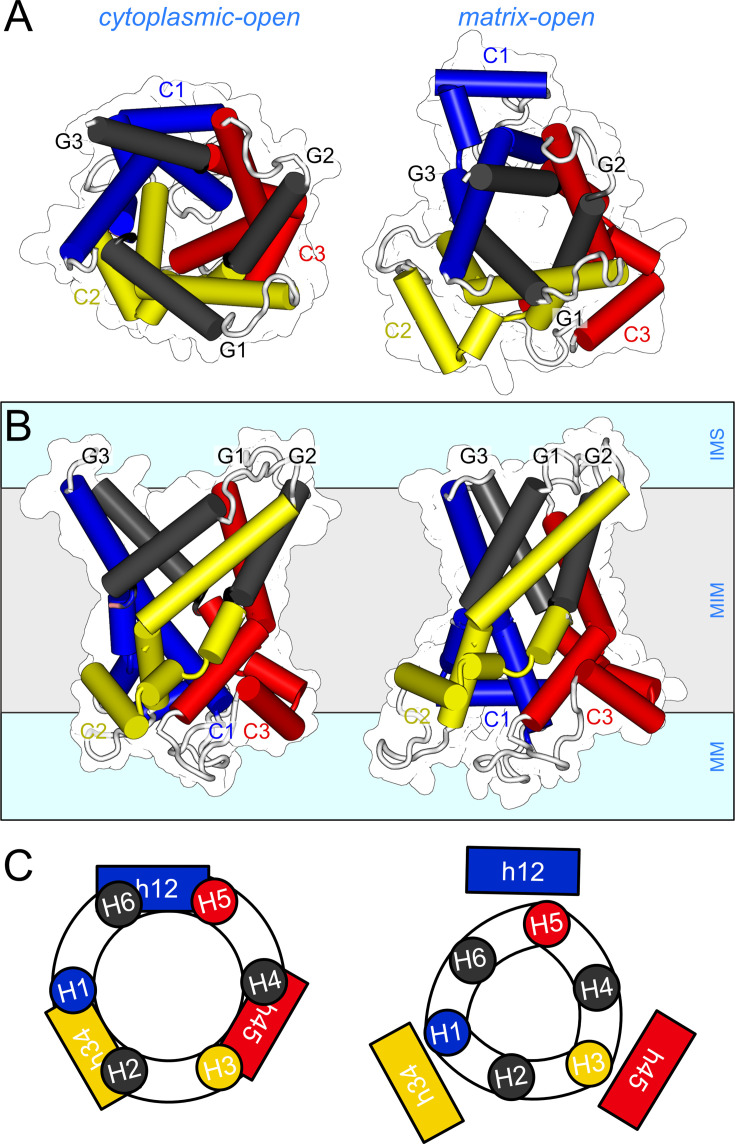
The structural mechanism of the mitochondrial ADP/ATP carrier. (**A**) Intermembrane space views and (**B**) lateral views of the cytoplasmic-open state (left) (PDB: 4C9H) [167]) and matrix-open state (right) (PDB: 6GCI) [168]. (**C**) Schematic representation of the helical rearrangements. Core elements C1, C2 and C3 are coloured in blue, yellow and red, respectively, and the gate elements G1, G2 and G3 in grey, with the helices indicated. IMS, intermembrane space; MIM, mitochondrial inner membrane; MM, mitochondrial matrix.

When the domain structures of the cytoplasmic-open and matrix-open states of the ADP/ATP carrier were compared, it became clear that each domain has two elements involved in the conformational changes [168]. Each domain consists of a *core element* (depicted in primary colours), comprises the odd-numbered helix, the matrix and linker helix, and the N-terminal part of the even-numbered helix, as well as a *gate element* (grey colours), comprising the C-terminal part of the even-numbered helix (Figure 6) [168]. The hinges between the core and gate elements double up as the contact points of the substrate-binding site [75,188,189], which enables the coupling of substrate binding to conformational changes (see further below). Thus, the conformational changes from the cytoplasmic-open to the matrix-open state involve outward movements of the three core elements and inward movements of the three gate elements, which occur concomitantly (Figure 6). The conformational changes from the matrix-open to the cytoplasmic-open state involve the same elements, but moving in reverse. Thus, the three core elements open and close access to the substrate-binding site from the mitochondrial matrix, whereas the three gate elements close and open access from the intermembrane space, respectively. Given that the hinge points also funcion as the substrate-binding site contact points [168,188,189], it is likely that substrate binding drives the conformational changes in all six elements simultaneously. A morphing trajectory between the two states is consistent with this mechanism, passing through an occluded intermediate state [168]. The three tightly bound cardiolipin molecules function as tethers, each linking two domains together [167,168,171,190–192].

**Figure 7:**
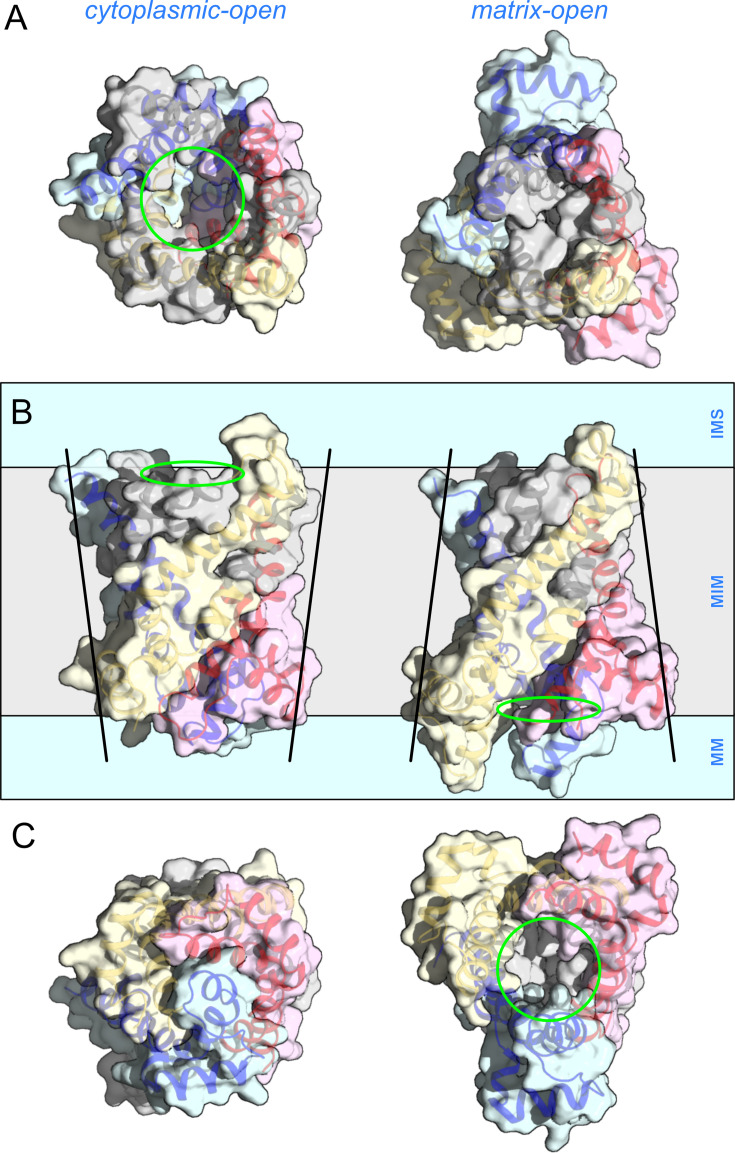
The surface changes in the transport cycle of the mitochondrial ADP/ATP carrier. (**A**) Intermembrane space views, (**B**) lateral views and (**C**) mitochondrial matrix views of the cytoplasmic-open state (left) (PDB: 4C9H) [163]) and matrix-open state (right) (PDB: 6GCI) [164]. Core elements 1, 2 and 3 are coloured in blue, yellow and red, respectively, and the gate elements 1, 2 and 3 in grey. The green circles indicate the accessible cavities with the substrate-binding site. IMS, intermembrane space; MIM, mitochondrial inner membrane; MM, mitochondrial matrix.

There are several aspects that are unique to this mechanism (Figure 6). First, the carrier mechanism exhibits three-fold pseudo-symmetry, mirroring the symmetry of its structure. Second, each transmembrane helix moves independently from the others, yet together they move in a co-ordinated manner to produce an alternating access mechanism. The individual helix mobility may explain why mitochondrial carriers are particularly susceptible to denaturation in detergent solutions [193–195]. In contrast, other transport mechanisms involve the concerted movement of transmembrane helices bundled together in a domain, although partial helical movements can also occur. Third, these conformational changes result in a dramatic rearrangement of the transmembrane helices—from a loose, circular configuration in the cytoplasmic-open state to a tight, triangular arrangement in the matrix-open state (Figure 6C). Fourth, the movements of the elements are accompanied by formation and disruption of a salt bridge network on either side of the carrier, called the matrix and cytoplasmic salt bridge network (Figure 7), which we will discuss in more detail later.

### Mitochondrial carriers function as monomers

For more than 45 years, proposals have been made for a dimeric organisation of mitochondrial carriers, initially for the bovine ADP/ATP carrier [196,197]. At that time, most transport proteins had 12 transmembrane helices, whereas mitochondrial carriers have only six transmembrane helices, which made it difficult to imagine how they could function as monomers, given the size of the transported substrates. Over this time period, many different techniques have been applied to indicate that mitochondrial carriers are dimers or tetramers, but we believe that in all of these cases analytical errors were made, as has been discussed previously [117,198]. When these factors are taken into account, it is far more likely that monomers rather than dimers were observed in each case. Recently, a new claim of a ‘transient’ dimer was made for SLC25A20 by using native gel electrophoresis with sarcosyl, a fairly harsh negatively charged detergent [199]. However, no correction was made for the binding of detergent and lipids to SLC25A20, which increases the particle size and alters its migration, unrelated to changes in oligomeric state, as has also been observed in blue native gel electrophoresis [200]. Also, no correction for bound detergent and lipid was made in size exclusion chromatography [199], which is required to obtain an accurate estimate of the protein size and weight [190,193]. In any case, it is highly unlikely that harsh detergents would preserve a few weak hydrophobic interactions between carriers; thus, we believe that also in this case only monomers were observed. Mitochondrial carriers in general can be cross-linked, but these experiments really show that their native forms are not cross-linked.

**Figure 8:**
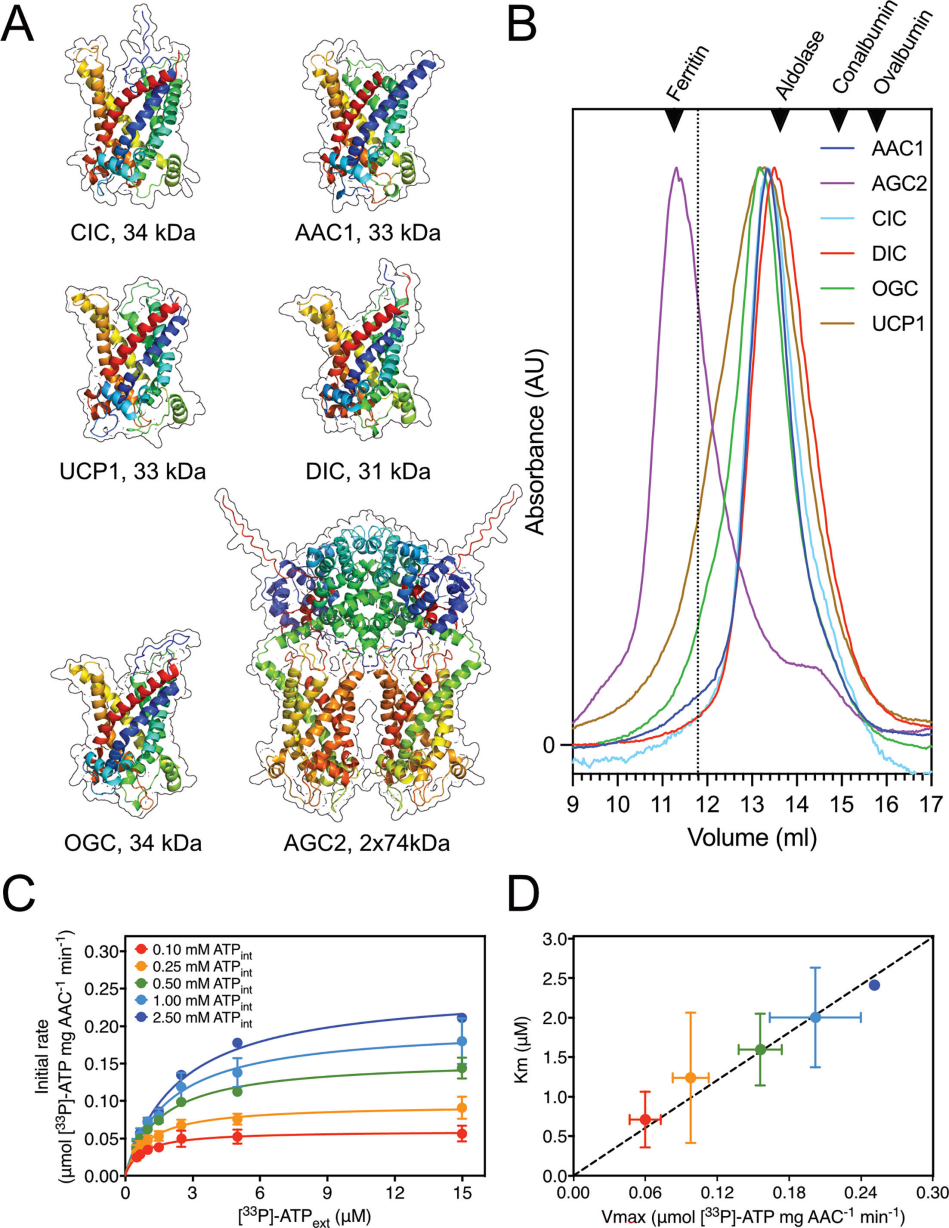
Oligomeric state and ping-pong kinetic mechanism**.** (**A**) Structural models of the human dicarboxylate carrier (DIC), oxoglutarate carrier (OGC), citrate carrier (CIC), ADP/ATP carrier (AAC1) and aspartate/glutamate carrier (AGC2) were all determined by the Alphafold 3.0 server [1], except for the experimentally determined structure of the uncoupling protein 1 (UCP1) (pdb entry: 8G8W) [2]. (**B**) Determination of the molecular weights in LMNG/TOCL by size exclusion chromatography. The normalised absorbance traces for monomeric AAC1 (blue), DIC (red), OGC (green), CIC (cyan), UCP1 (brown) and dimeric AGC2 (purple) are shown. The standards used for sizing were ferritin (440 kDa), aldolase (158 kDa), conalbumin (76 kDa) and ovalbumin (43 kDa). The dotted line indicates the elution volume of a hypothetical dimer peak for DIC, OGC and CIC. Data from [203]. (**C**) Two-substrate transport analysis catalysed by the mitochondrial ADP/ATP carrier. Michaelis–Menten plots of 0.10 mM (red traces), 0.25 mM (orange traces), 0.50 mM (green traces), 1.00 mM (light blue traces) and 2.50 mM (dark blue traces) internal ATP concentrations[204]. (**D**) Cimadamore-Werthein/King plot, where the independently determined *Km* values are plotted against the Vmax values for the various internal substrate concentrations. The kinetic parameters were determined by fitting the Michaelis–Menten curves through iteration (same colour scheme as in **C**).

Previously, a dimeric state for the bovine ADP/ATP carrier was proposed based on native mass spectrometry [201], but the protein had the incorrect native mass and lacked the association of tightly bound cardiolipin molecules, a key feature of mitochondrial carriers [166–168,171,174]. Recently, using the same technique, the yeast ADP/ATP carrier Aac2p was investigated, and this time the correct mass was identified, showing that the carrier is a monomer with tightly bound cardiolipins with different chain lengths [202] in agreement with earlier observations [167,168,171,190–192]. Thus, with the advantage of hindsight, the ‘bovine dimer’ might have been a simple case of mistaken identity, as there are better candidates for the observed mass in the mitochondrial proteome, which are present as dimers. To date, the only confirmed dimers in the SLC25 family are the mitochondrial aspartate/glutamate carriers (SLC25A12 and SLC25A13), which associate via an additional N-terminal domain, but the two carrier domains do not interact with each other [3] and transport independently of each other [203]. *De novo* predictions by Alphafold 3.0 [1] provide further support for the notion that the mitochondrial aspartate/glutamate carriers are indeed structural dimers, whereas other mitochondrial carriers are structural monomers (Figure 8A).

The best arguments against a dimeric state come from understanding the structural properties and mechanism of mitochondrial carriers. First, the surface interacting with the core of the membrane consists almost entirely of aliphatic or aromatic residues, which can only provide weak and non-specific interactions (Figure 4F). Second, the three-dimensional shape of the carrier changes profoundly during every stage of the transport cycle, meaning there is no stable interaction interface (Figure 7). Third, the surface composition also changes as the gate elements slide across the core elements, as some residues change position from being external to being interhelical and back (Figure 5). Fourth, the carrier does not protrude significantly beyond the surface of the membrane [205], and the hydrophilic surfaces also change drastically during the transport cycle (Figure 6). The loop regions are highly variable in length and composition, and thus, they do not provide stable and conserved interaction opportunities either. Fifth, the surface is coated with the acyl chains of the tightly bound cardiolipin, which will prevent the carriers from directly interacting with other carriers and other proteins in the high-density inner membrane, so they can move freely through their conformational changes. Most importantly, the carriers have all the required mechanistic and structural elements to function as monomers, as we will see next, and thus, there is no selective advantage to form dimers.

In the high-density inner membrane, a diverse set of mitochondrial carriers can be found in different conformational states at any moment, making it highly unlikely that they can form stable dimers or have lasting interactions with mitochondrial carriers or other proteins. At best, they may form weak, transient and non-specific interactions that are of no functional consequence.

## The strong interaction networks involved in the alternating access mechanism

**Figure 9:**
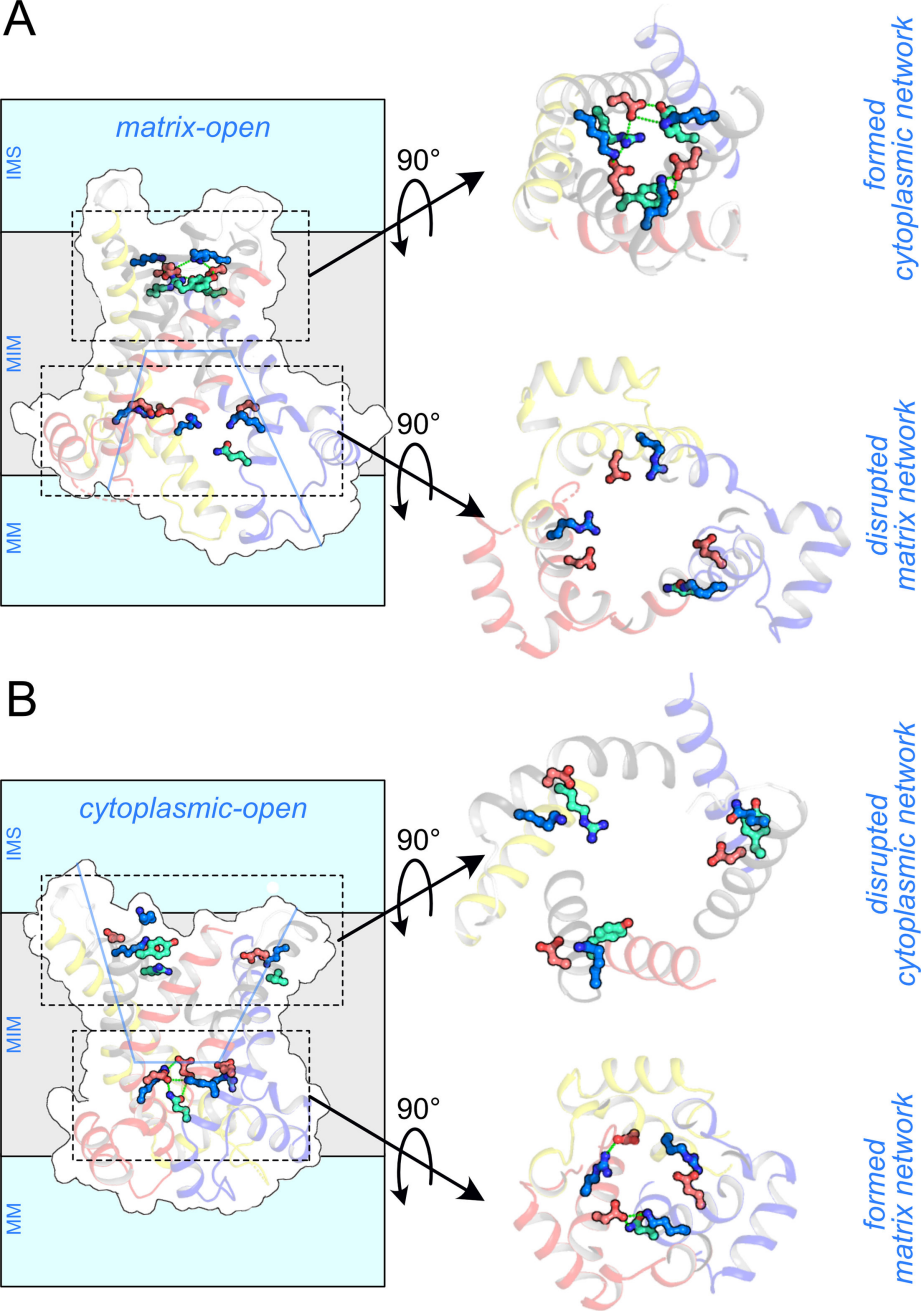
The properties of the matrix and cytoplasmic salt bridge networks and braces. Lateral views of the (**A**) matrix-open state (PDB: 6GCI) [168] and (**B**) cytoplasmic-open state (PDB: 4C9H) [167]. Core elements 1, 2 and 3 are coloured in blue, yellow and red, respectively, and the gate elements 1, 2 and 3 in grey. The negatively and positively charged residues of the cytoplasmic network (top right) and matrix network (bottom right) are shown in red and blue, respectively. The glutamine and tyrosine braces are shown in green cyan. The approximate position of the central water-filled cavity is indicated with a blue line. IMS, intermembrane space; MIM, mitochondrial inner membrane; MM, mitochondrial matrix.

The three odd-numbered transmembrane helices (H1, H3 and H5) of the core elements contain highly conserved Px[DE]xx[RK]xxxQ motifs on the matrix side of the carrier (Figure 9). In the cytoplasmic state, the proline kinks bring the C-terminal ends of the odd-numbered helices together in the centre of the carrier. The negatively charged and positively charged residues of these motifs form the matrix salt bridge network [75,206], as observed in the structure of the cytoplasmic-open state [166,167]. Glutamine residues, which are positioned one helical turn below, act as a brace of the salt bridge interactions of the matrix network, referred to as glutamine braces [167]. The mitochondrial ADP/ATP carriers have only one glutamine brace, but other carriers may have up to three [167], which contribute to the overall interaction energy of the matrix network. Beneath the C-terminal ends of the odd-numbered helices lies a set of structurally important residues [207], which together with the matrix network and glutamine braces, form the matrix gate.

The three even-numbered helices (H2, H4 and H6) on the gate elements contain highly conserved [YF][DE]xx[RK] motifs on the cytoplasmic side of the carrier [75] (Figure 9). In the matrix-open state, the negatively and positively charged residues of this motif form the cytoplasmic salt bridge network [75,168,208]. The hydroxyl group of the preceding tyrosine residue forms hydrogen bond interactions with the negatively charged residue of the neighbouring domain [168], which is called the tyrosine brace, in analogy to the glutamine brace of the matrix network. Mitochondrial carriers have one to three of these tyrosine braces, modulating the overall interaction energy of the cytoplasmic network. Underneath is a set of aromatic and hydrophobic residues (the hydrophobic plug) that together with the cytoplasmic network and tyrosine braces form the cytoplasmic gate (Figure 9).

In conclusion, when the carrier changes conformation from the cytoplasmic-open to the matrix-open state, the three core elements move outwards, disrupting the matrix salt bridge network and glutamine braces, whereas the three gate elements move inwards, resulting in the formation of the cytoplasmic salt bridge network and tyrosine braces (Figure 9). The converse happens when the carrier moves from the matrix-open to the cytoplasmic-open state.

The unusually strong interaction networks of the mitochondrial carriers have probably evolved for several reasons: (i) the strong binding energy of the charged substrates [209], (ii) the requirement for tightly sealed gates in the presence of a high membrane potential to prevent proton and ion leak [166–168], (iii) the need for equimolar exchange of substrates, preventing conformational changes without substrate binding, and (iv) the requirement for equal occupancies of the matrix-open and cytoplasmic-open states to maximise the exchange rates of substrates [75].

## The highly charged substrate-binding sites of mitochondrial carriers

Several computational approaches have been applied to locate the substrate-binding site of SLC25 mitochondrial carriers. The first approach used chemical and distance constraints to identify residues capable of distinguishing amino acid, keto acid and adenine nucleotide substrates [188,189]. A central substrate-binding site was identified, which is composed of contact points on the even-numbered transmembrane helices. We have proposed that contact point II on transmembrane H4 can discriminate between different chemical classes of substrates (e.g. keto or amino acids), contact point I on transmembrane H2 can distinguish between different substrates within the same chemical class, whereas contact point III on transmembrane H6 is nearly always positively charged, irrespective of the substrate [188,189]. Subsequently, it was shown that these contact points are also the hinge points between the core and gate elements of each domain, linking substrate binding to conformational changes and the formation and disruption of the two salt bridge networks [168]. The second approach exploited the three-fold pseudo-symmetrical properties of mitochondrial carriers [165], consisting of three homologous sequence repeats [164], indicating that the ancestral protein was likely to be fully symmetric. However, most substrates are asymmetric, having different chemical groups, and thus, an asymmetric binding site has evolved to bind them. A search procedure was employed to identify asymmetric and symmetric residues for all human and yeast mitochondrial carriers [75]. In each case, a cluster of asymmetric residues was identified in the central cavity that are complementary to the chemical properties of the substrates [165]. Some of them may have evolved to provide direct interactions with the substrates, but others to allow the binding of the substrates by generating a suitable pocket [75]. By and large, molecular dynamics studies are in agreement, showing that electrostatic interactions are a major driving force for substrate binding [53,172,173,210]. Mutagenesis studies, which determine the effect of a specific mutation on transport, are also broadly in agreement (reviewed in [64,211–213]). However, the problem is that a negative effect on transport can have many possible causes, such as impairment of targeting, insertion and folding of the protein or impairment of the transport mechanism, due to loss of conformational dynamics, network formation or substrate binding. Direct experimental proof for the role of a residue in substrate binding using transport assays can be obtained by showing that its mutation causes a change in substrate specificity. This approach was used to show that a lysine residue in contact point II of the human mitochondrial ornithine carriers (SLC25A2 and SLC25A15) was crucial to its substrate specificity [122]. When lysine was mutated to an arginine residue, the substrate specificity was extended from just ornithine to other basic amino acids, and when lysine was mutated to a glutamine residue, the enantiomer specificity was expanded to include D-isoforms as well [122].

**Figure 10:**
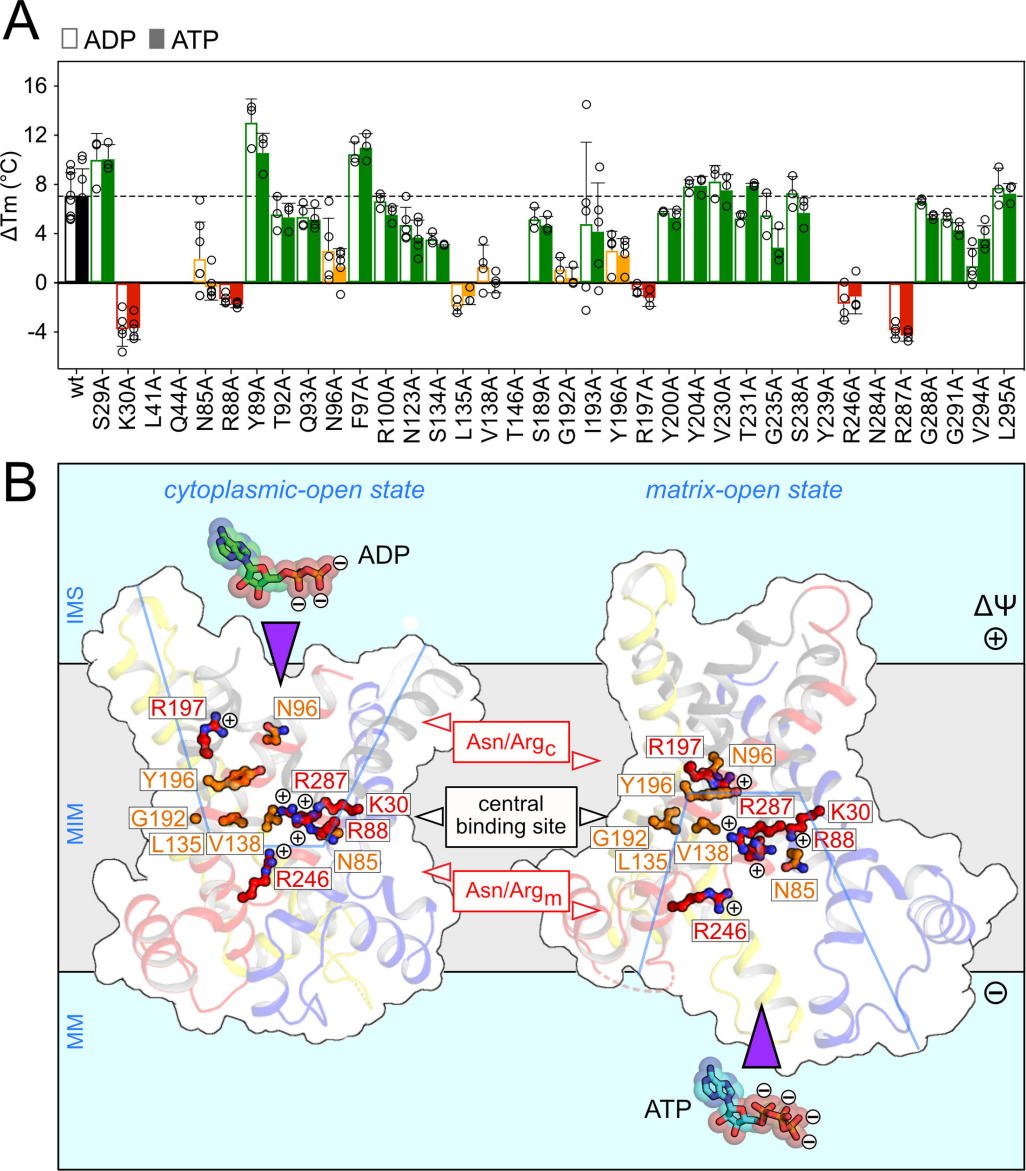
Substrate-binding residues in the central cavity of the mitochondrial ADP/ATP carrier. (**A**) Thermostability shift values (ΔTm) measured for 10 mM ADP (empty bars) or ATP (filled bars). Variants that are not significantly different from wildtype ADP/ATP carrier of *Thermothelomyces thermophila* (TtAac) are shown in green, whereas variants of critical and important residues are shown in red and orange, respectively. No significant differences in the shifts between ADP and ATP were observed for the wildtype and variants. Data taken from Mavridou et al. [207]. (**B**) Essential (red) and important (orange) residues involved in ADP and ATP binding. Lateral views of the cytoplasmic-open state [ScAac2, PDB code 4c9h, chain A] (left) and matrix-open state (TtAac, PDB code 6gci, chain A) (right) of the mitochondrial ADP/ATP carrier. The residue numbering is from TtAac. The approximate position of the central water-filled cavity is indicated with a blue line. MM, mitochondrial matrix; MIM, mitochondrial inner membrane; IMS, mitochondrial intermembrane space.

In a second approach, we have used substrate-induced thermostability shifts to provide experimental evidence for residues involved in substrate binding. A thermostability shift assay monitors the unfolding of a protein population in a temperature ramp by thiol labelling, e.g.,with the CPM probe [214,215], or by changes in intrinsic tryptophan/tyrosine fluorescence, e.g., nano-differential scanning fluorimetry (nanoDSF) [61,216]. We have shown that this approach can be used to map the binding sites of specific inhibitors of the mitochondrial ADP/ATP carrier, carboxy-atractyloside and bongkrekic acid, in good agreement with the known structures [167,168,207]. Evidence for all types of bond interactions can be obtained, whether they are ionic, polar, aromatic or hydrophobic interactions. We have also shown that binders cause a significant thermostability shift compared with other chemically related molecules, providing a new method to screen for candidate substrates and coupling ions for members of three different transporter (super)families [217]. Since the thermostability shift is specific for substrates, it also provides an approach to identify the residues involved in substrate binding, as was done for adenine nucleotide binding to the mitochondrial ADP/ATP carrier [207]. Of the 36 mutated residues of the translocation pathway, only eleven were found to be involved in substrate binding. Single alanine replacements of five positively charged residues caused a complete abolishment of the shift (Figure 10A), indicating that they are essential for binding. They are thought to interact with the negatively charged phosphate moieties of the nucleotides. Another six caused a significant reduction of the shift (Figure 10A), indicating that they are involved in binding of the adenosine moiety jointly. Most importantly, the shifts for ADP and ATP were, within error, the same, indicating that both nucleotides bind to the same set of residues with the same chemistry (Figure 10A). Based on their locations, the residues can also be divided into three sets. The first set, the central substrate-binding site, does not alter much between the two known states (Figure 10B), meaning that the substrate binds to this site when the carrier transitions between states. The same site had been identified in the chemical/distance searches [188,189], symmetry analysis [75], state-dependent site accessibility [166–168] and molecular dynamics simulations [53,172,173,210]. Unexpectedly, two separate pairs of asparagine/arginine residues were also identified, i.e., one pair on the cytoplasmic side (Asn/Arg_c_) and one pair on the matrix side (Asn/Arg_m_) of the central binding site, which are involved in substrate binding in a state-dependent manner (Figure 10B). In the cytoplasmic-open state, the residues of the Asn/Arg_c_ are pointing towards the opening of the cavity, whereas they are part of the central binding site in the matrix-open state. Conversely, the residues of the Asn/Arg_m_ pair are part of the central binding site in the cytoplasmic-open state, whereas they are pointing towards the opening of the cavity in the matrix-open state. These two pairs are likely to be responsible for the initial binding and final release of the nucleotides, whilst also controlling the geometry of nucleotide binding [207]. These observations indicate that the adenine nucleotides move through the carrier in a step-wise manner, explaining why in principle ADP and ATP can be transported in either direction, fully reversibly.

## Mitochondrial carriers have a ping-pong kinetic mechanism

The structural and functional analyses are consistent with the mitochondrial carriers existing and functioning as monomers and having a single binding site for both exchanged substates. These observations indicate that the mitochondrial carriers have an alternating access mechanism, consistent with ping-pong or double displacement kinetics in which one substrate is imported first and released from the central binding site before counter-substrate binds and is exported. However, it has been previously proposed that mitochondrial carriers operate with a simultaneous or sequential kinetic mechanism (reviewed in [218] and references in [203,204]), where the transport protein binds both exchanged substrates at the same time, forming a ternary complex, which in our view is incompatible with the proposed structural mechanism.

Thus, we have recently revisited the two-substrate kinetics of several SLC25 mitochondrial carriers: the mitochondrial citrate carrier (SLC25A1), ADP/ATP carrier (SLC25A4), dicarboxylate carrier (SLC25A10), oxoglutarate carrier (SLC25A11) and aspartate/glutamate carrier (SLC25A13) [203,204]. All of these carriers are monomeric apart from the aspartate/glutamate carrier, which is dimeric, as shown by Alphafold 3.0 modelling (Figure 8A) and size exclusion chromatography (Figure 8B) [203,204], by taking into account the amount of bound lipid/detergent [190,193]. For this analysis, purified carriers were reconstituted into liposomes, allowing full control of the substrate gradients for the kinetic analysis. For several internal concentrations of unlabelled substrate, we measured the initial exchange rates for a range of external radiolabelled substrate concentrations, yielding two-substrate kinetics. Initial uptake rates were obtained by generating complete uptake curves using robotics, meaning that all recorded data points were generated under identical conditions. Uptake curves were fitted with a model that determined the initial rates accurately. For the kinetic analysis, Michaelis–Menten curves were generated for all internal substrate concentrations, as shown here for the mitochondrial ADP/ATP carrier (Figure 8C). When the kinetic data were visualised as Lineweaver–Burk plots, the lines corresponding to different internal concentrations appeared to be parallel [203,204], which is a hallmark of a ping-pong kinetic mechanism [219]. A more accurate approach is to determine the apparent Km and Vmax values by iteration and to plot the kinetic parameters against each other (Figure 8D) [203]. A linear fit of these data showed that the *Km*/Vmax ratio for all of the measured internal concentrations was similar for all internal concentrations in the case of the mitochondrial ADP/ATP carrier (SLC25A4) (Figure 8D) [204]. The same observations were made for all other carriers, demonstrating that they all had ping-pong or double displacement kinetics [203]. An interesting case is the mitochondrial aspartate/glutamate carrier, which is a structural dimer [3], but functions with a ping-pong kinetic mechanism [203] because the carrier domains function independently of each other [3]. Together with the earlier observation for the mitochondrial carnitine/acylcarnitine carrier (SLC25A20) [220], it is now likely that all mitochondrial carriers operate with a ping-pong kinetic mechanism. This is consistent with the notion that they function as monomers with an alternating access mechanism. Thus, the kinetic mechanism of SLC25 mitochondrial carriers is not exceptional at all, as all other transport proteins are likely to have an alternating access/ping-pong mechanism, despite having very different structural mechanisms (Figure 5).

## Unexpected charge movements during exchange of negatively charged substrates

A direct consequence of the ping-pong kinetic mechanism of the mitochondrial ADP/ATP carrier [204] is that ADP^3-^, carrying three negative charges, needs to be imported against the membrane potential, before ATP^4-^, carrying four negative charges, can be exported with the membrane potential. In principle, moving the three negative charges against the membrane potential would be highly endergonic, preventing import. Thus, the key outstanding question was how the mitochondrial ADP/ATP carrier, and other anion exchangers, accomplish this remarkable feat to achieve high directional exchange rates despite this energetic barrier [221].

**Figure 11:**
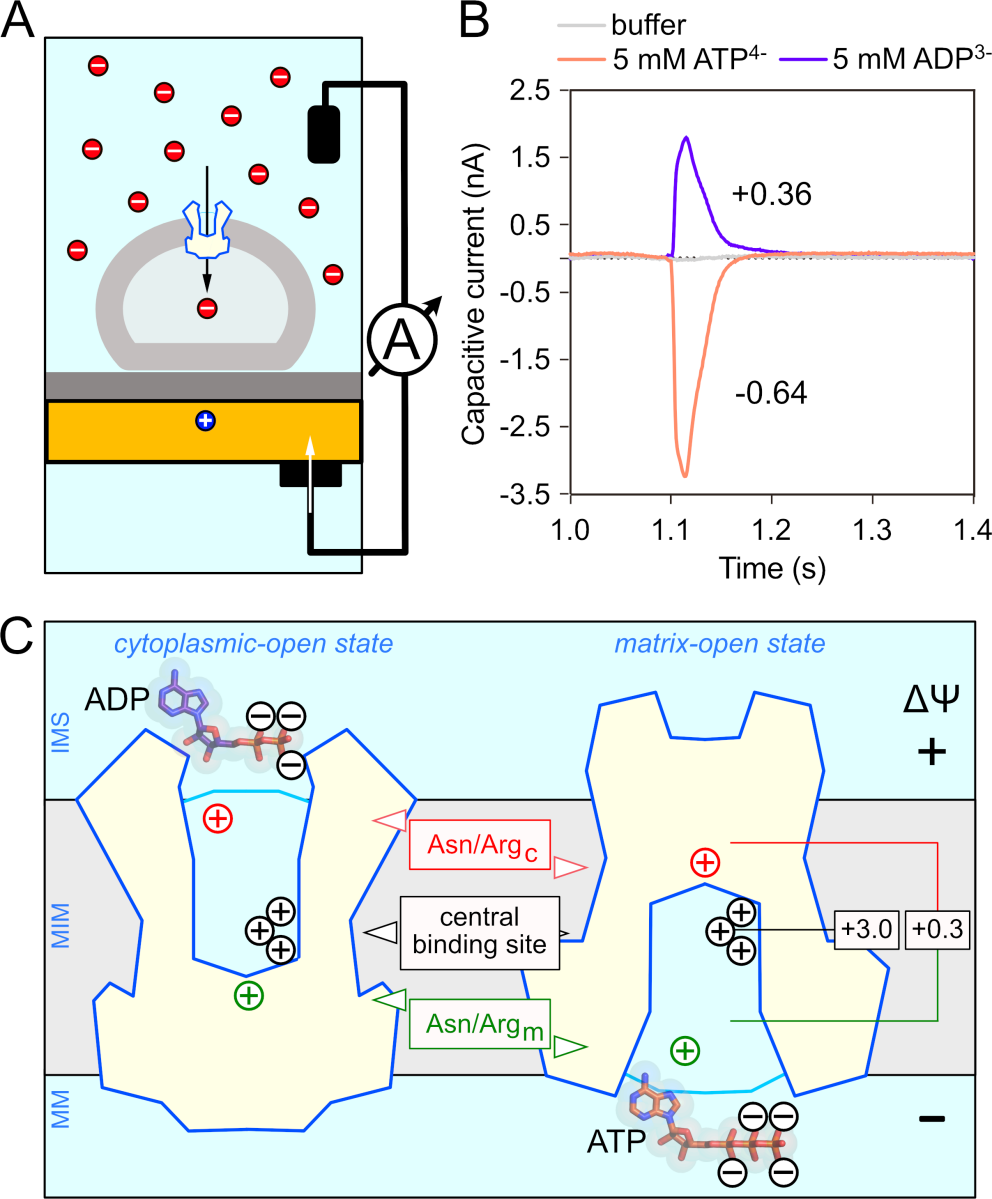
Charge movements by the mitochondrial ADP/ATP carrier. (**A**) Schematic of the experimental setup for measuring charge movements using solid-supported membrane-based electrophysiology. (**B**) Capacitive currents induced by 5 mM ADP or ATP, measured using empty liposomes (no internal substrate) in which the wildtype ADP/ATP carrier was reconstituted. The curves represent one example experiment, with each current being recorded twice [221]. (**C**) Schematic representation of the positions of the five positive charges of the substrate-binding site in the cytoplasmic-open (left) and matrix-open state (right), essentially as in Figure 10B. Indicated are the three positively charged residues of the central substrate-binding site, which should account for the +3.0-charge movement from one side of the membrane to the other (full translocation). Also indicated are the positive charges of the Asn/Arg pair on the cytoplasmic side (Asn/Arg_c_) (red) and matrix side (Asn/Arg_m_) (green), which move from the water phase to the protein phase and vice versa, accounting for the partial +0.3-charge movement (partial translocation).

To understand charge translocation during adenine nucleotide exchange, we reconstituted the mitochondrial ADP/ATP carrier into liposomes and measured the capacitive currents generated by ADP^3-^ or ATP^4-^ import into proteoliposomes by using solid supported membrane-based electrophysiology, which was pioneered by the Bamberg laboratory (Figure 11A) [222–224]. In this technique, a capacitive current is measured, which is not a physical current moving across the membrane, but instead, the product of the capacitance and the rate of change of the membrane potential. A membrane potential can be generated either by a charge moving through the membrane (transport) or by changing the dielectric around a fixed charge (binding). The capacitive current can be integrated over time to give the charge movement, which can be calibrated against a known charge. It is well established that only the unprotonated species are transported by the ADP/ATP carrier [138,223,224] and that there is no proton movement upon transport [225].

As expected, ATP^4-^ import generated a negative capacitive current, meaning a negative charge movement (Figure 11B) [221]. However, the ADP^3-^ import generated a positive capacitive current, indicating a net positive charge movement, even though the substrate has three negative charges (Figure 11B) [221]. The integration of the capacitive current over time and the formal charge difference between ADP^3-^ and ATP^4-^ can be used to quantify the charge movements. In this way, it can be shown that a transport step of ADP^3-^ results in a +0.3-charge movement, whereas a transport step of ATP^4-^ results in a −0.7-charge movement. These observations are best explained by assuming that the carrier moves +3.3 charges across the membrane concomitantly with the substrate, as for ADP^3-^ this gives +3.3–3.0 = +0.3 charge movement and for ATP^4-^ +3.3–4.0 = −0.7 charge movement. This interpretation is consistent with earlier observations [222,223], indicating that these charge movements are universal for ADP/ATP carriers. As discussed earlier, five positively charged residues are involved in substrate binding; three positively charged residues are in the central substrate-binding site, whereas another two are part of the Asn/Arg_c_ and Asn/Arg_m_ pairs (Figure 10B). The most likely candidates for +3.0-charge movements are the three positively charged residues in the central substrate-binding site. These residues are initially located within a water-filled cavity exposed to one side of the membrane and move with the substrate to a cavity exposed to the other side. As such, they are fully translocated across the dielectric barrier of the membrane (Figure 11C). The movements of the Asn/Arg_c_ and Asn/Arg_m_ pairs are likely to be responsible for the +0.3-charge movements, as these integer charges only move through a fraction of the membrane dielectric (Figure 11C). Single alanine replacement mutants confirmed the involvement of all five positively charged residues, as ADP^3-^ transport induced a negative charge movement [221].

**Figure 12:**
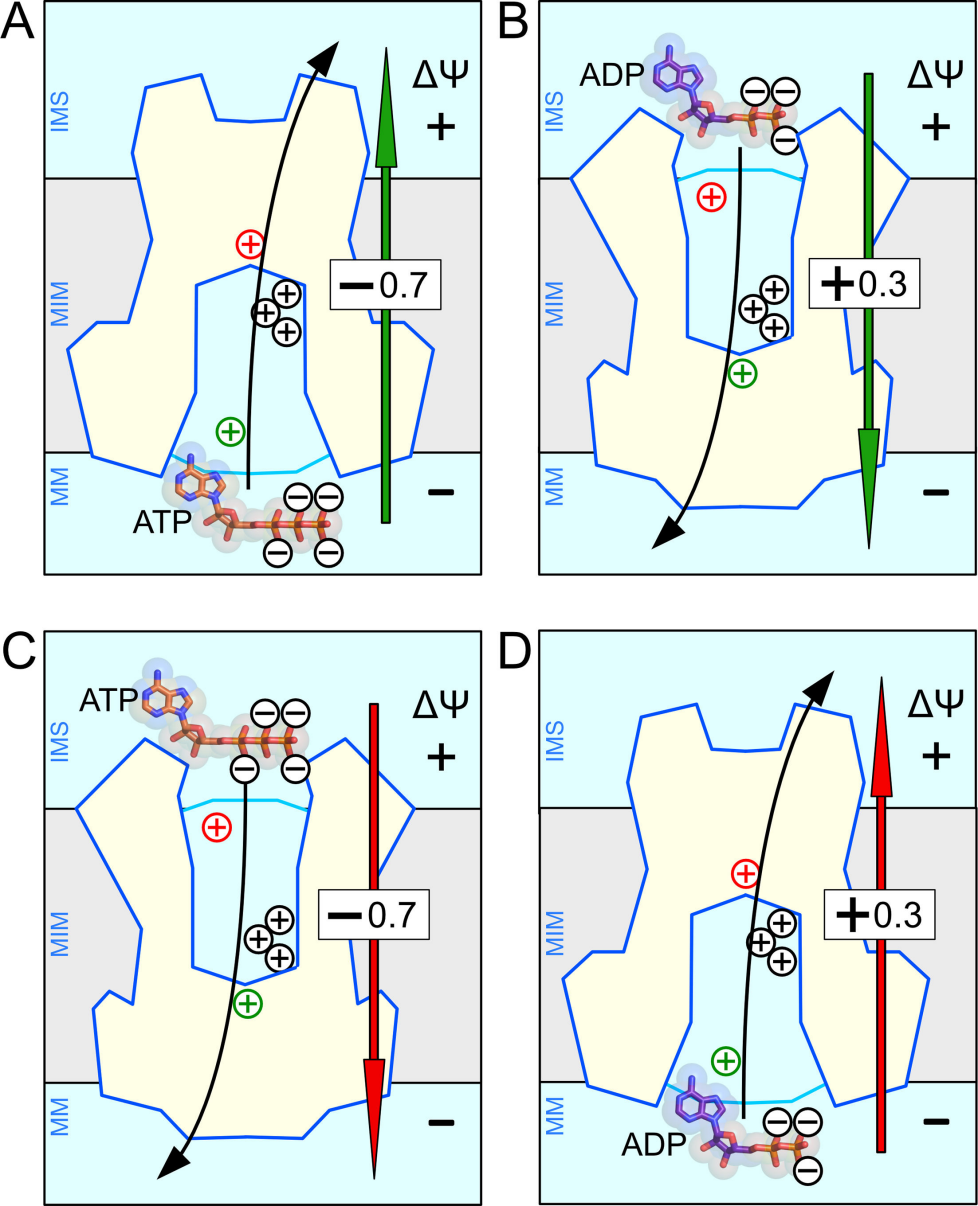
Overall charge movements dictate the directionality of adenine nucleotide transport in the presence of a membrane potential. (**A**) Export of ATP and (**B**) import of ADP transport, steps that are both stimulated by the membrane potential, as the overall charge movement moves with the membrane potential. (**C**) Import of ATP and (**D**) export of ADP, transport steps that are both opposed by the membrane potential, as the overall charge movement moves against the membrane potential. The negatively charged ADP^3-^ and ATP^4-^ are shown in purple and salmon red, respectively, whereas the positively charged residues of the central binding site ( + 3.0 movement) and the Asn/Arg_c_ and Asn/Arg_m_ pairs (+0.3 movement) are shown in black, red and green plus symbols, respectively. The straight arrows show the direction of overall charge transport, either stimulated (green) or opposed (red) by the membrane potential.

Mitochondrial carriers are fully reversible molecular machines [209], meaning that any substrate can be transported in either direction across the membrane. The carriers have a single central substrate-binding site, residues that guide the substrates in and out, as well as gates on either side of the protein, explaining why there is no directionality encoded within the structural mechanism. However, in the context of a functional mitochondrion, the charge movements have important implications for the net directionality of substrate transport. When ATP^4-^ is exported, −0.7 charge is overall moved along the electric field of the membrane potential (positive outside), thereby facilitating this transport step (Figure 12A). More remarkably, when ADP^3-^ is imported, a net +0.3 charge is moved along the electric field (negative inside) and thus is also stimulated by the membrane potential, rather than being opposed (Figure 12B). Thus, the positively charged residues have evolved to allow both transport steps to be driven by the membrane potential, stimulating the exchange of adenine nucleotides. Typically, cytosolic ATP concentrations are maintained high, whereas ADP levels are kept low, to drive ATP hydrolysis for energy-requiring metabolic processes. If the carrier is fully reversible, why are substantial amounts of ATP not transported back into mitochondria? There are enzymes in the vicinity that convert ATP to ADP to maintain substrate concentration gradients, but that is not the complete picture. ATP import would result in a net -0.7 charge movement against the membrane potential and would therefore be strongly disfavoured (Figure 12C). Similarly, phosphate and ADP concentrations are maintained at high levels in the mitochondrial matrix to drive ATP synthesis, implying that ADP could in principle be exported. However, ADP^3-^ export would result in a net +0.3 charge displacement against the electric field and would therefore be opposed by the membrane potential (Figure 12D). Thus, in the context of a functional mitochondrion, ADP^3-^ import and ATP^4-^ export are both stimulated and ADP^3-^ export and ATP^4-^ import are both inhibited by the membrane potential. The effect of the membrane potential is to apply a small Newtonian force in each transport step, meaning that substrates, going in the reverse direction, are pushed back, whereas substrates, going in the forward direction, are propelled forwards. In this way, the mitochondrial ADP/ATP carriers achieve high and directional adenine nucleotide exchange rates across the mitochondrial inner membrane to fuel the energy-requiring processes of the eukaryotic cell.

What would happen if the membrane potential were to collapse due to failure of the respiratory chain? ATP synthesised by glycolysis would then be imported down its concentration gradient, with each transport step resulting in a net −0.7-charge entering the mitochondrion, contributing to the build-up of a membrane potential. ADP, generated by hydrolysis, would be then exported, also contributing to the buildup of a membrane potential by the movement of a +0.3-charge out of the mitochondrion. Thus, in this way, the mitochondrial ADP/ATP carrier would maintain a membrane potential, stimulating mitochondrial protein import and molecule transport, and would also provide glycolytic ATP for energy-requiring mitochondrial functions, such as mitochondrial DNA replication and protein synthesis. Given the properties of the substrate-binding sites of mitochondrial carriers [75,188,189], it is clear that similar charge movements must be part of their fundamental transport mechanism, but they need further experimental exploration.

## The transport cycle of the mitochondrial ADP/ATP carrier

**Figure 13:**
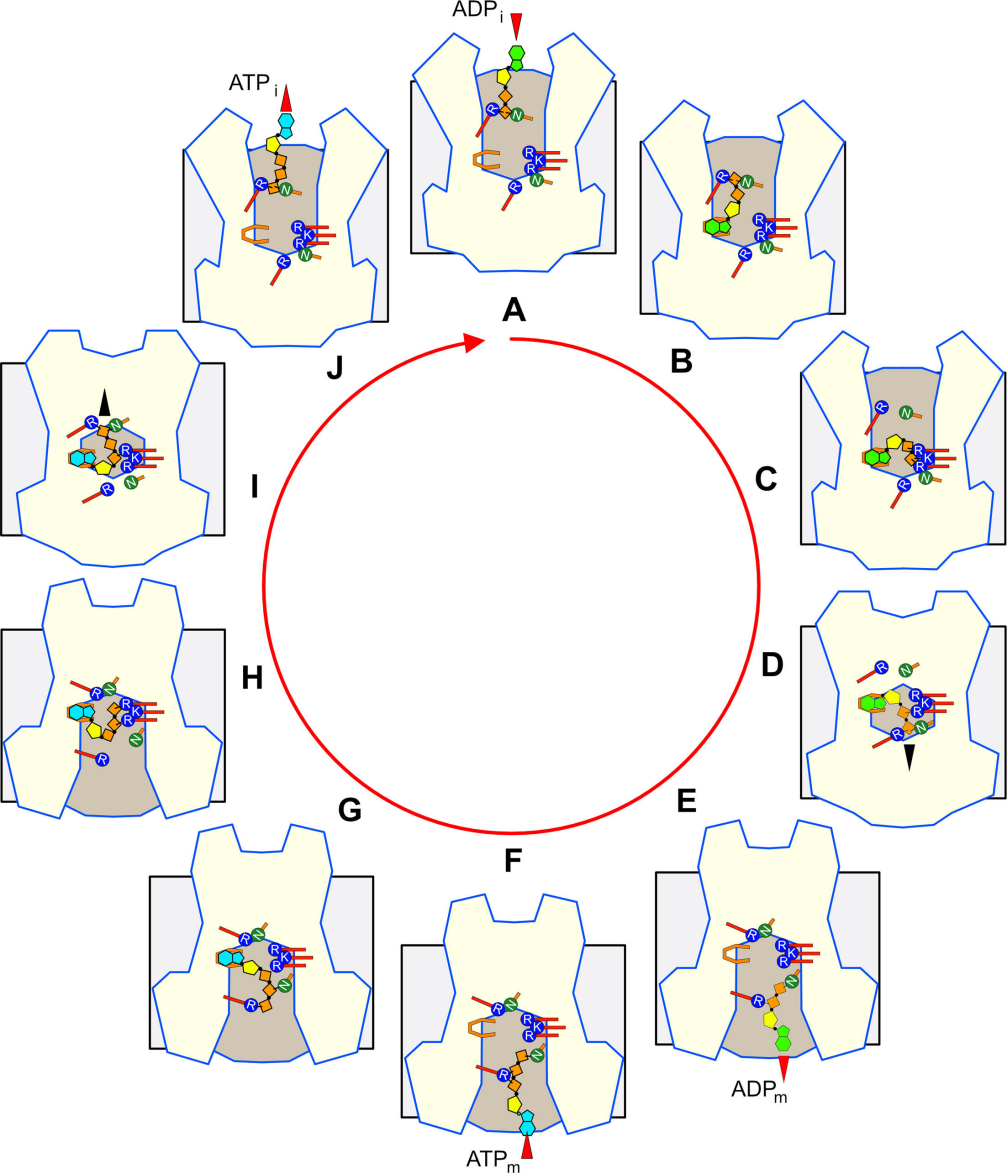
Schematic representation of the substrate-binding events as part of the transport cycle of the mitochondrial ADP/ATP carrier. Substrate interactions at different stages of the transport cycle.(**A**) Phosphate moieties of ADP interacting with Asn/Arg_c_, (**B**) adenine group binding, (**C**) phosphate moieties binding to central site, (**D**) ADP-bound occluded state, (**E**) ADP leaving via Asn/Arg_m_, (**F**) ATP interacting with Asn/Arg_m_, (**G**) adenine group binding, (**H**) phosphate moieties binding to central site, (**I**) ATP-bound occluded state and (**J**) ATP leaving via Asn/Arg_c_. The substrates ADP and ATP have an adenine moiety (green and cyan, respectively), a ribose moiety (yellow) and two or three phosphate moieties (orange), respectively. The adenosine binding site is represented by a horseshoe, whereas positively charged and polar residues of the binding site are shown in blue and green, respectively. The black arrowheads show the substrate movements that induce the conversion between states, whereas the red ones indicate entry and exit of the substrates. All steps are fully reversible, but in the presence of the membrane potential they follow the direction A to J.

The various pieces of the puzzle can now be combined in order to provide a coherent model of the transport cycle of the mitochondrial ADP/ATP carrier (Figure 13). First, by Brownian motion, ADP enters the cavity of the carrier in the cytoplasmic state, and its phosphate moieties bind electrostatically to Asn/Arg_c_, which they encounter first (Figure 13A). Once bound, the hydrophobic adenine moiety of ADP binds to the central hydrophobic/aromatic binding site residues (Figure 13B). This is an important step to test the specificity, as mitochondrial ADP/ATP carriers transport only ADP, ATP and their deoxy-variants, but not AMP, GDP or GTP [53,138]. Once the adenine moiety is bound, the negatively charged phosphate moieties of ADP bind to the positively charged residues of the central binding site, neutralising its charge to lower the energy barrier (Figure 13C). All contact points of the binding site are now engaged, allowing progression to the occluded state (Figure 13D). The formation of the cytoplasmic salt bridge network allows progression to the matrix-open state (Figure 13E), where binding to the Asn/Arg_m_ pair leads to release of ADP into the mitochondrial matrix for ATP synthesis (Figure 13E). The newly synthesised ATP is transported out of the mitochondrion in a similar series of interactions and conformational changes, but occurring in reverse (Figure 13F-J). We propose that all other members of the SLC25 mitochondrial carrier family operate using a similar mechanism but adapted to their specific requirements.

## Future work

Many questions with regard to the physiological role, function and mechanism of SLC25 mitochondrial carriers remain unanswered. First, our understanding of their role in mitochondrial and cellular physiology and disease is still incomplete. For example, when and where different isoforms are expressed in relation to their physiological role is not fully understood. Also, some members of the SLC25 mitochondrial carrier family in humans have not been functionally characterised, while others have not been identified. Conversely, there are many molecules known to traverse the mitochondrial inner membrane, but they have not been assigned to specific transport proteins. How pathogenic mutations lead to dysfunctional carriers and how they, in turn, lead to pathophysiological processes in neuromuscular, metabolic and developmental disorders requires further investigation (reviewed in [7,9]). Importantly, the use of mitochondrial carriers as potential drug targets to treat specific pathologies is only beginning to be explored.

Then there are many open questions with regard to their molecular mechanism. The transport mode of many SLC members is still undefined, i.e. whether they operate by antiport, symport or uniport mechanisms. How substrate recognition is achieved at the exclusion of other substrates is not well defined. How is substrate binding coupled to conformational changes in the matrix and cytoplasmic state transitions? Some carriers carry out substrate symport with protons or other ions, but how is this coupling achieved? These are just some of the many outstanding issues of this fascinating, but very peculiar, transporter family.

## Abbreviations

AAC: ADP/ATP carrier
ETF: electron transfer flavoprotein
PNC: pyrimidine nucleotide carrier
PNC: pyrimidine nucleotide carrier
SLC25: solute carrier family 25
